# Supplementary material for: Activation of Nrf2 Signaling Augments Vesicular Stomatitis Virus Oncolysis via Autophagy-Driven Suppression of Antiviral Immunity
Source: Mol Ther. 2017 May 17;25(8):1900–16. doi: 10.1016/j.ymthe.2017.04.022 (PMC5542709; doi:10.1016/j.ymthe.2017.04.022)
Supplement: Document S1. Figures S1–S7 [file mmc1.pdf]

YMTHE, Volume *25*

Supplemental Information

Activation of Nrf2 Signaling Augments Vesicular Stomatitis Virus Oncolysis via Autophagy-Driven Suppression of Antiviral Immunity

David Olagnier, Rassin R. Lababidi, Samar Bel Hadj, Alexandre Sze, Yiliu Liu, Sharadha Dayalan Naidu, Matteo Ferrari, Yuan Jiang, Cindy Chiang, Vladimir Beljanski, Marie-Line Goulet, Elena V. Knatko, Albena T. Dinkova-Kostova, John Hiscott, and Rongtuan Lin

Supplementary Materials

Supplemental Figure Legends

Figure S1. Sulforaphane potentiates VSVΔ51 infectivity in various cancer cell lines. Human prostate DU145, human breast MDA-MB, and murine breast TS-A were pretreated for 24h with SFN and then challenged with VSVΔ51. 24 hours later, viral infectivity was determined by flow cytometry. Data are the means ± SEM from at least two experiments performed in duplicate or triplicate on each cell line.

Figure S2. Sulforaphane treatment does not affect PC-3 viability *in vitro.* Cytotoxic effect of SFN alone was tested in PC-3 cells by treating the cells with increasing concentrations of SFN for 24h and 48h. **(a)** SFN toxicity on PC-3 cells was visualized 48h after treatment by light microscopy. **(b-c)** The percentage of viable and apoptotic cells was assessed by flow cytometry using a 7AAD/annexin-V staining as previously described.

Figure S3. Sulforaphane does not increase VSVΔ51 infectivity and oncolysis in non-cancerous human fibroblasts. (a-b) Human prostate PC-3 cancer cells and human MRC-5 normal fibroblasts were pre-treated with SFN (15µM) for 24h and were subsequently infected with VSVΔ51-GFP (MOI 0.1). Infectivity **(a)** and oncolytic activity **(b)** were determined by flow cytometry at 48h post-infection. Data are the means ± SEM from two independent experiments performed in quadruplicate for MRC-5 and triplicate for PC-3.

Figure S4. **Diethylmaleate stimulates Nrf2 promoter activity and increases VSVΔ51 infectivity in resistant prostate cancer cells. (a)** HEK 293T cells were pre-treated for 24h with increasing doses of diethylmaleate (DEM) and ARE promoter activity was assessed using a luciferase assay. **(b)** PC-3 cells were pre-treated with sulforaphane (SFN) (20µM) or diethylmaleate (100µM) for 24h and were subsequently infected with VSVΔ51-GFP (MOI 0.1 or 1). Viral infectivity was determined by flow cytometry based on GFP expression.

Figure S5. SFN activates Nrf2 and increases VSVΔ51 infectivity independently of ROS. (a) ROS generation in SFN-treated PC-3 cells was monitored by flow cytometry using CM-H2DCFDA (1µM) at 4 and 24h following treatment. H_2O_2 (500µM) and pyocyanin (100µM) were used as positive internal controls of ROS accumulation. **(b)** ROS generation was also monitored 24h after treatment with H_2O_2 (500µM) or pyocyanin (100µM) in presence or absence of L-NAC (2mM) and SFN (20µM). **(c)** HEK 293T cells were pre-treated with L-NAC (10mM) for 30 min which was subsequently washed away. Cells were then treated with SFN (10µM) for 18h. ARE promoter activity was assessed using a luciferase assay. **(d)** PC-3 cells were pre-treated with L-NAC (10mM) for 30 min. L-NAC was removed and cells stimulated or not with SFN (20µM) for 24h prior to challenge with VSVΔ51 (MOI 1) for 24h. Viral infectivity was determined by flow cytometry based on GFP expression.

Figure S6. IRF3 does not bind to DNA following SFN stimulation in VSVΔ51-infected PC-3 cells. IRF3-nuclear binding activity was assessed using an IRF3-binding ELISA TransAM kit after treatment of PC-3 cells with SFN (20µM) for 24h and infection with VSVΔ51 (MOI 1) for 8 hrs. The data are expressed as relative arbitrary units. Data are the means from two independent experiments.

Figure S7. Abrogating autophagy reduces VSVΔ51 infection and replication in A549 lung cancer cells. A549 cells were transfected with control or Atg7 siRNA and 48h later were challenged with VSVΔ51-GFP (MOI 0.01) for an extra day. (**a**) VSVΔ51 infection was determined by flow cytometry and pictures were taken using the ZOE fluorescent cell imager. Data are the means± SEM from three independent experiments. (**b**) Viral replication was assessed by plaque assay. Data are the means± SEM from one experiment performed in triplicate.

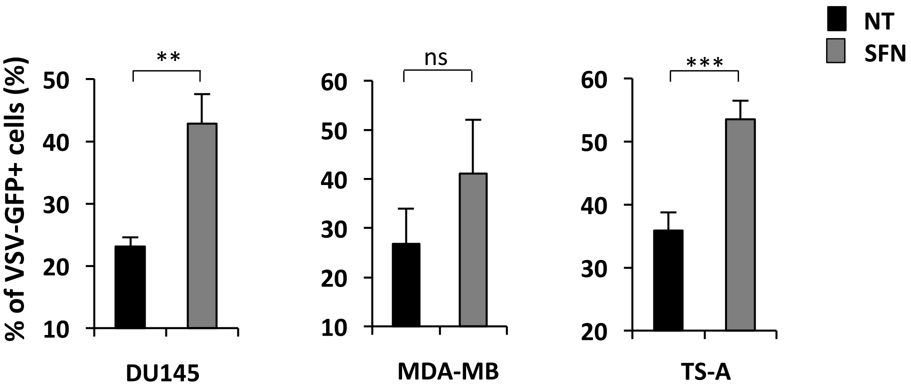
NT
SFN
% of VSV-GFP+ cells (%)
**
ns
***
50
40
30
20
10
60
50
40
30
20
10
60
50
40
30
20
DU145
MDA-MB
TS-A

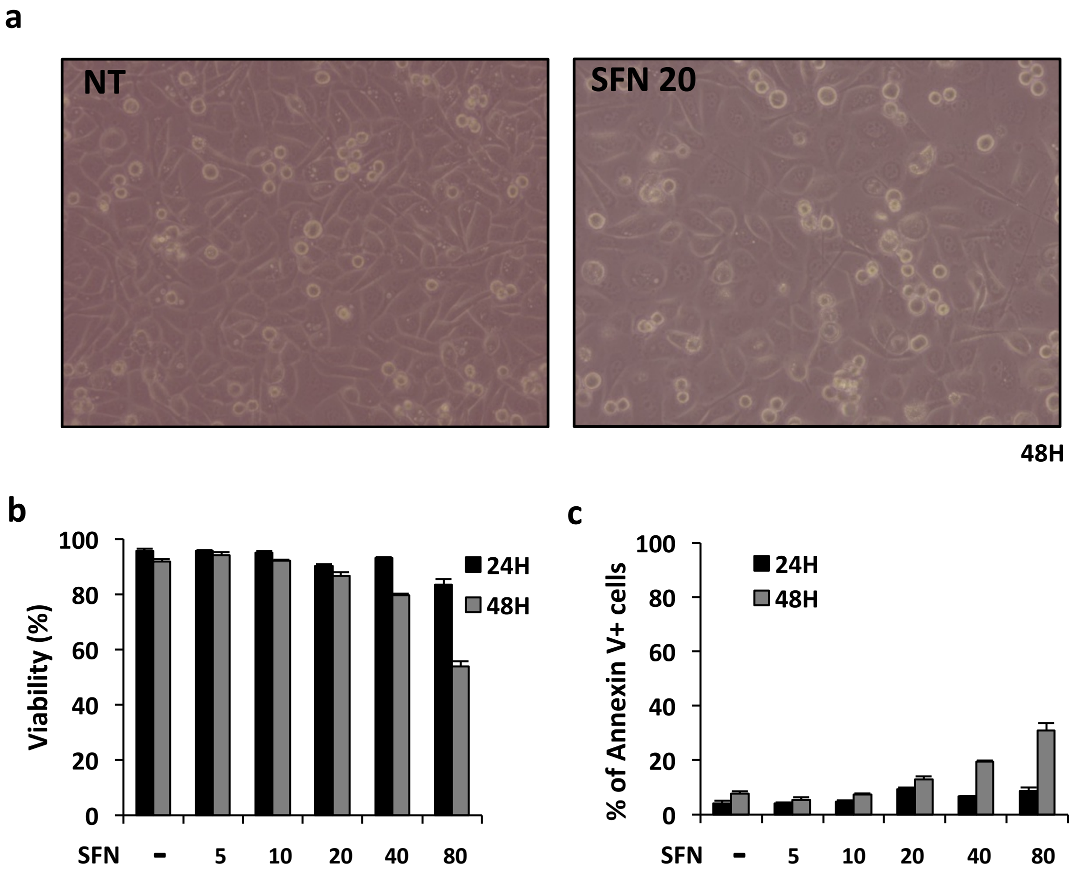
a
NT
SFN 20
48H
b
Viability (%)
100
80
60
40
20
0
24H
48H
SFN
-
5
10
20
40
80
c
% of Annexin V+ cells
100
80
60
40
20
0
24H
48H
SFN
-
5
10
20
40
80

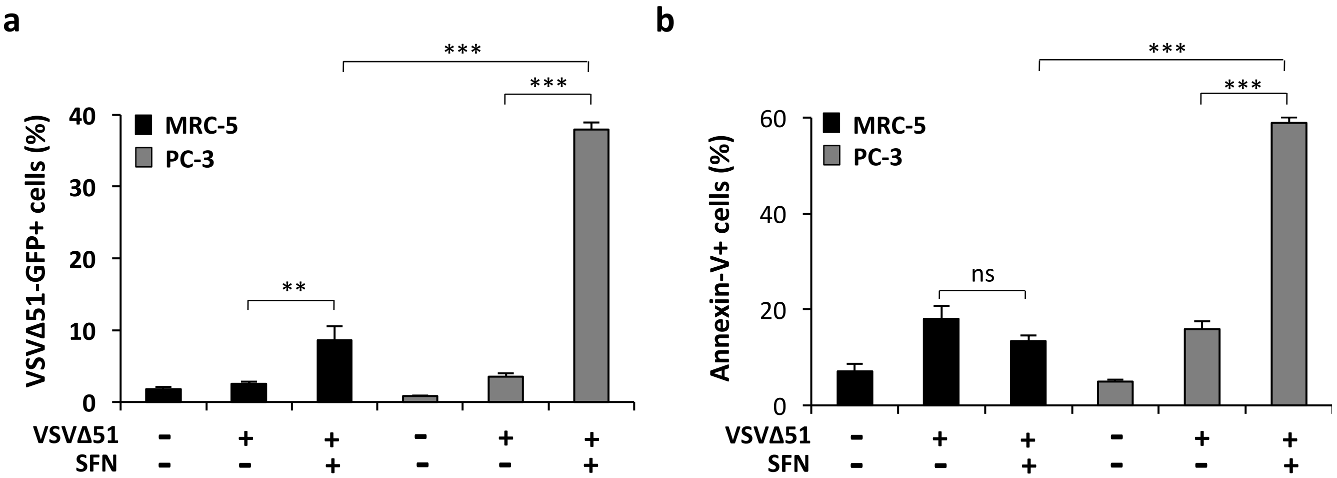

a
VSVΔ51-GFP+ cells (%)
40
30
20
10
0
MRC-5
PC-3
**
***
***
VSVΔ51
SFN
b
Annexin-V+ cells (%)
60
40
20
0
MRC-5
PC-3
ns
***
***
VSVΔ51
SFN

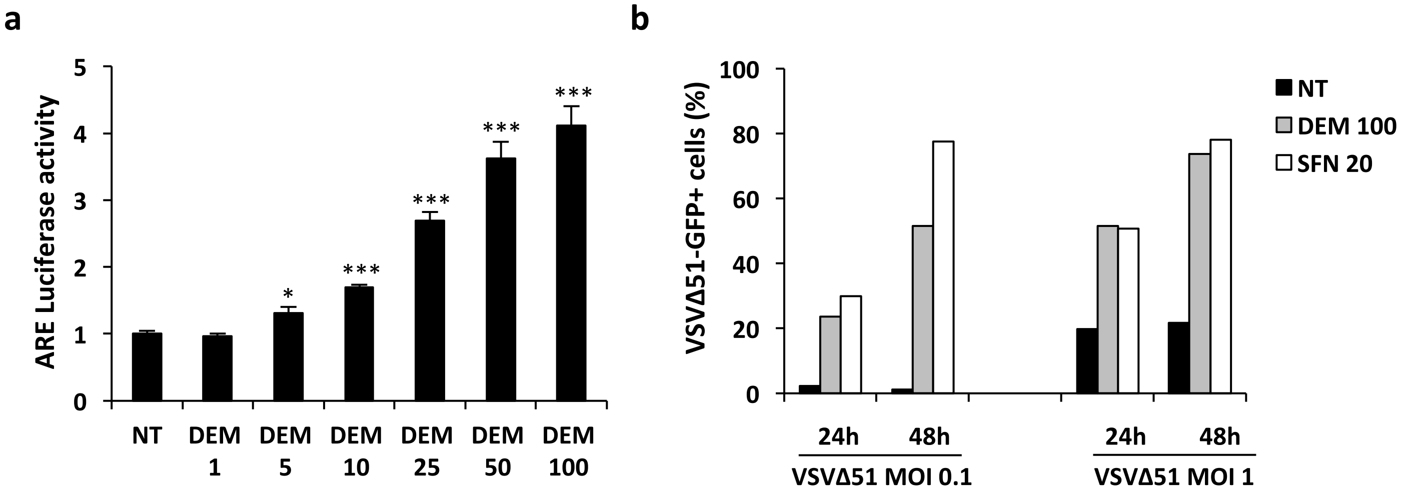

a
ARE Luciferase activity
0
1
2
3
4
5
*
***
***
***
***
NT
DEM 1
DEM 5
DEM 10
DEM 25
DEM 50
DEM 100
b
VSVΔ51-GFP+ cells (%)
0
20
40
60
80
100
NT
DEM 100
SFN 20
24h
48h
VSVΔ51 MOI 0.1
24h
48h
VSVΔ51 MOI 1

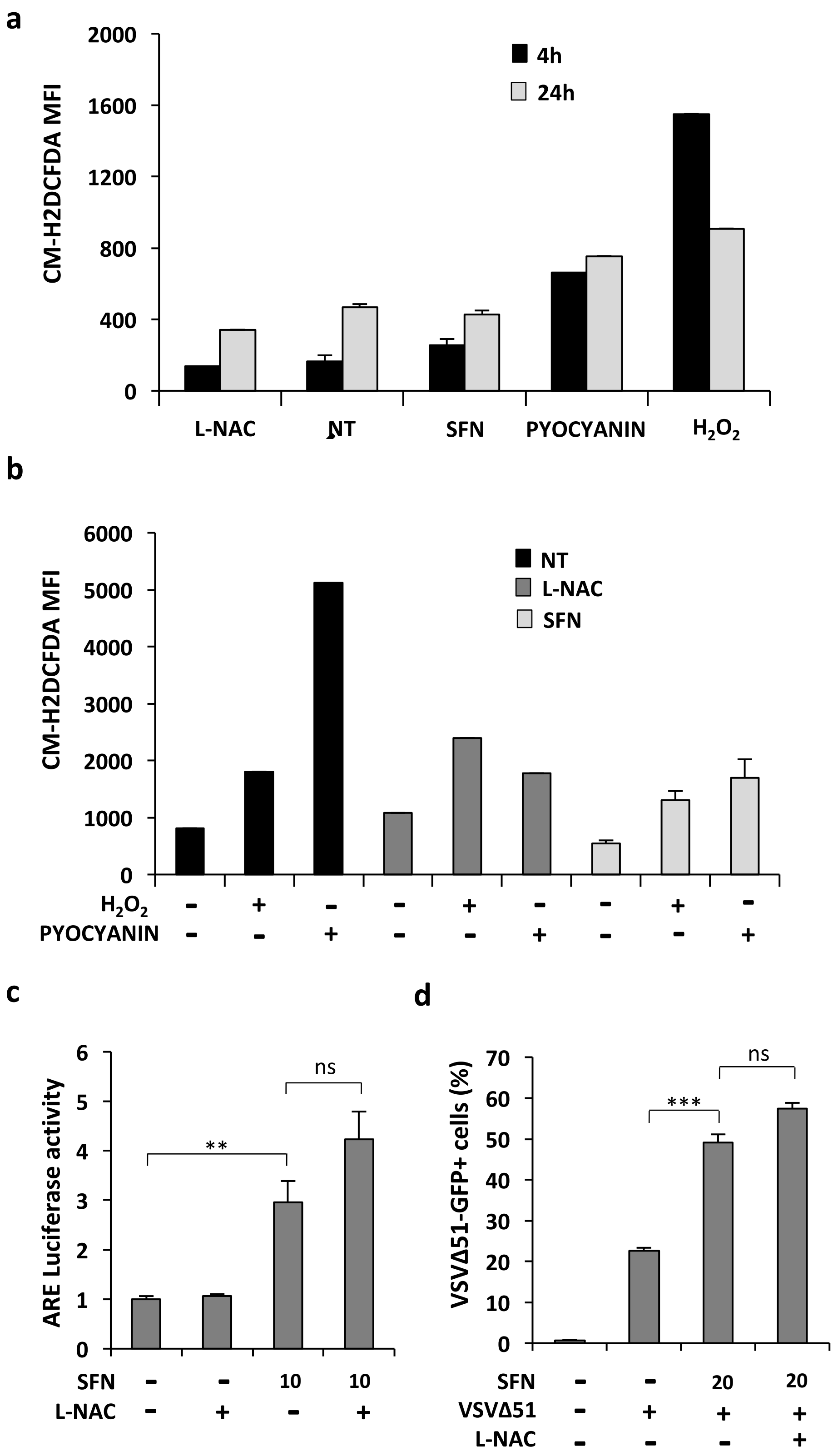
a
CM-H2DCFDA MFI
4h
24h
L-NAC
NT
SFN
PYOCYANIN
H2O2
b
CM-H2DCFDA MFI
NT
L-NAC
SFN
H2O2
PYOCYANIN
c
ARE Luciferase activity
ns
**
SFN
L-NAC
d
VSVΔ51-GFP+ cells (%)
ns
***
SFN
VSVΔ51
L-NAC

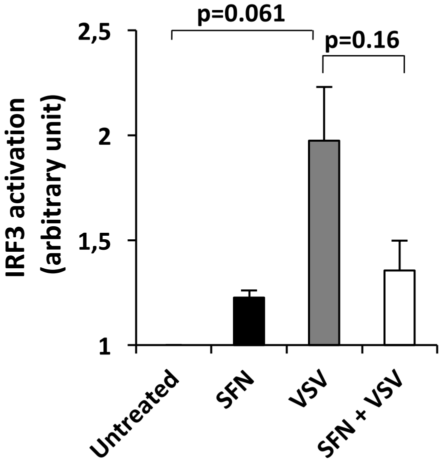

p=0.061
p=0.16
2,5
2
1,5
1
IRF3 activation
(arbitrary unit)
Untreated
SFN
VSV
SFN + VSV

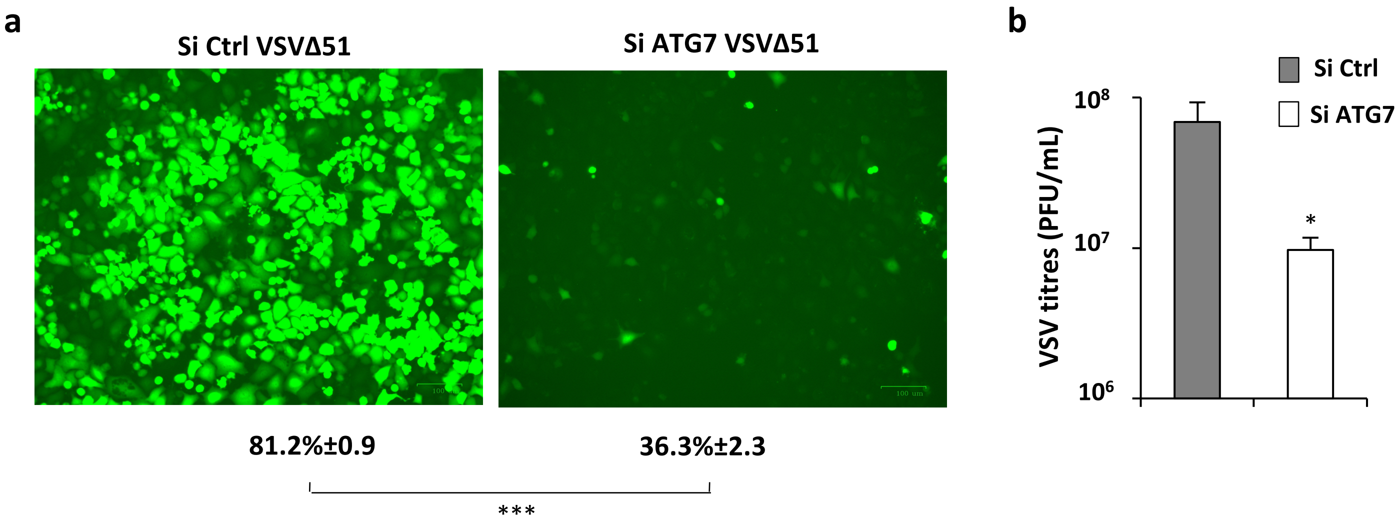
a
Si Ctrl VSVΔ51
Si ATG7 VSVΔ51
81.2%±0.9
36.3%±2.3
***
b
Si Ctrl
Si ATG7
VSV titres (PFU/mL)
10^8
10^7
10^6
*
