# Supplementary material for: Activation of Nrf2 Signaling Augments Vesicular Stomatitis Virus Oncolysis via Autophagy-Driven Suppression of Antiviral Immunity
Source: Mol Ther. 2017 May 17;25(8):1900–16. doi: 10.1016/j.ymthe.2017.04.022 (PMC5542709; doi:10.1016/j.ymthe.2017.04.022)
Supplement: Document S2. Article plus Supplemental Information [file mmc2.pdf]

Molecular Therapy
Original Article

Activation of Nrf2 Signaling Augments Vesicular Stomatitis Virus Oncolysis via Autophagy-Driven Suppression of Antiviral Immunity

David Olagnier,[1,2,10] Rassin R. Lababidi,[1,2,3,9] Samar Bel Hadj,[1,9] Alexandre Sze,[1,2] Yiliu Liu,[1,2] Sharadha Dayalan Naidu,[4] Matteo Ferrari,[8] Yuan Jiang,[1,2] Cindy Chiang,[5] Vladimir Beljanski,[6] Marie-Line Goulet,[1] Elena V. Knatko,[4] Albena T. Dinkova-Kostova,[4,7] John Hiscott,[8] and Rongtuan Lin[1,2,3]

[1]Lady Davis Institute-Jewish General Hospital, McGill University, Montreal, QC H3T 1E2, Canada; [2]Division of Experimental Medicine, McGill University, Montreal, QC H4A 3J1, Canada; [3]Department of Microbiology & Immunology, McGill University, Montreal, QC H3A 2B4, Canada; [4]Jacqui Wood Cancer Centre, Division of Cancer Research, School of Medicine, University of Dundee, Dundee DD1 9SY, UK; [5]Department of Microbiology, University of Chicago, Chicago, IL 60637, USA; [6]NSU Cell Therapy Institute, Nova Southeastern University, Fort Lauderdale, FL 33314, USA; [7]Department of Pharmacology and Molecular Sciences and Department of Medicine, Johns Hopkins University School of Medicine, Baltimore, MD 21205, USA; [8]Pasteur Laboratory, Istituto Pasteur-Fondazione Cenci Bolognetti, Rome 00161, Italy

Oncolytic viruses (OVs) offer a promising therapeutic approach to treat multiple types of cancer. In this study, we show that the manipulation of the antioxidant network via transcription factor Nrf2 augments vesicular stomatitis virus Δ51 (VSVΔ51) replication and sensitizes cancer cells to viral oncolysis. Activation of Nrf2 signaling by the antioxidant compound sulforaphane (SFN) leads to enhanced VSVΔ51 spread in OV-resistant cancer cells and improves the therapeutic outcome in different murine syngeneic and xenograft tumor models. Chemoresistant A549 lung cancer cells that display constitutive dominant hyperactivation of Nrf2 signaling are particularly vulnerable to VSVΔ51 oncolysis. Mechanistically, enhanced Nrf2 signaling stimulated viral replication in cancer cells and disrupted the type I IFN response via increased autophagy. This study reveals a previously unappreciated role for Nrf2 in the regulation of autophagy and the innate antiviral response that complements the therapeutic potential of VSV-directed oncolysis against multiple types of OV-resistant or chemoresistant cancer.

INTRODUCTION

Oncolytic virotherapy is an innovative form of experimental cancer therapy that uses different virus platforms as self-amplifying biotherapeutics to selectively infect, multiply, and kill cancer cells while sparing healthy tissues.[1–4] The therapeutic potential of oncolytic viruses (OVs) is highlighted by the approval of oncolytic recombinant herpes virus talimogene laherparepvec (T-VEC) to treat malignant melanoma in the United States.[5] However, variability in the clinical response to OV-based therapies and engagement of the adaptive immune response against viral rather than tumor antigens are critical roadblocks to the large-scale implementation of OVs against multiple types of cancer.[6] In addition, nearly one-third of cancer cell lines and many primary tumors maintain a residual innate immune response that renders cells resistant to infection and virus-mediated cell killing.[6] To circumvent these issues, several strategies that combine OVs with chemical agents or engineering recombinant viruses with enhanced antitumoral activity have been investigated.[7–14]

Vesicular stomatitis virus (VSV), an enveloped negative-sense RNA virus from the *Rhabdoviridae* family, is a prototypical OV that has demonstrated potent oncolytic activity in preclinical models and is being evaluated in clinical trials.[6,15,16] Different genetic variants of VSV have been engineered to preferentially target tumors without compromising healthy cells. For example, VSVΔ51 contains a deletion at methionine 51 in the matrix protein that improves its tumor specificity and impairs its replication in normal cells that have functional antiviral defenses.[17,18] In previous studies, we demonstrated the synergistic effect of different agents, including histone deacetylase inhibitors (HDIs), as chemical switches to dampen the type I interferon (IFN) response and to increase VSVΔ51 replication within resistant malignancies.[10,12] We also showed that pharmacologic disruption of the BCL-2-Beclin-1 interactions facilitated autophagy and increased the VSVΔ51-mediated cytolytic effect in chronic lymphocytic leukemia cells.[19]

Nuclear factor erythroid 2-related factor 2 (Nrf2) is a transcriptional regulator involved in the maintenance of redox homeostasis through

Received 12 December 2016; accepted 24 April 2017;
http://dx.doi.org/10.1016/j.ymthe.2017.04.022.

[9]These authors contributed equally to this work.

[10]Present address: Department of Biomedicine, Aarhus Research Center for Innate Immunology, Aarhus University, Aarhus C 8000, Denmark

Correspondence: David Olagnier, Department of Biomedicine, Aarhus Research Center for Innate Immunology, Aarhus University, Aarhus C 8000, Denmark.
E-mail: olagnier@biomed.au.dk

Correspondence: John Hiscott, Istituto Pasteur-Fondazione Cenci Bolognetti, Rome 00161, Italy.
E-mail: john.hiscott@istitutopasteur.it

Correspondence: Rongtuan Lin, Lady Davis Institute-Jewish General Hospital and Division of Experimental Medicine, McGill University, Montreal, QC H3T 1E2, Canada.
E-mail: rongtuan.lin@mcgill.ca

 © 2017 The Author(s).
This is an open access article under the CC BY license (http://creativecommons.org/licenses/by/4.0/).

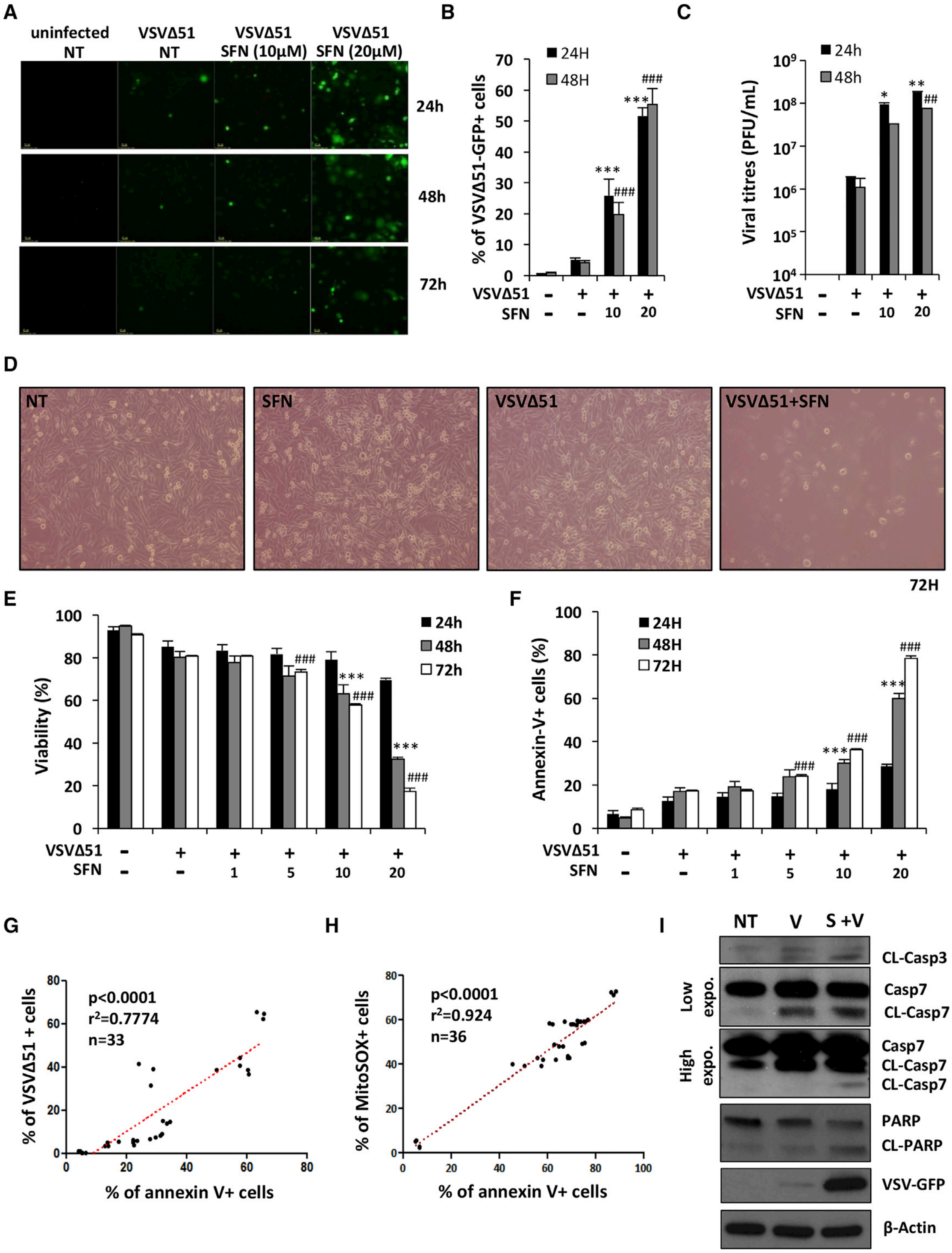

Figure 1. Sulforaphane Enhances VSVΔ51-Mediated Oncolytic Activity in Resistant PC-3 Cells

PC-3 cells were pretreated for 24 hr with increasing concentrations of sulforaphane (SFN) and were subsequently infected with VSVΔ51-GFP (MOI 1). (A and B) Infectivity was determined by fluorescent microscopy (A) or quantified by flow cytometry (B) at the indicated times. (C) Viral replication was assessed by plaque assay. (D) Cell survival was imaged by light microscopy 72 hr following VSVΔ51 challenge. (E and F) The percentage of viable (E) and apoptotic cells (F) was assessed using an annexin-V/7AAD staining by flow cytometry at the indicated times. (A–F) Data are the means ± SEM from two experiments performed in triplicate. (G) The correlation between the percentage of VSVΔ51-GFP^{+}

(legend continued on next page)

the control of basal and induced expression of an array of antioxidant enzymes.[20] Under homeostatic conditions, Nrf2 binds to Kelch-like ECH-associated protein 1 (Keap1), a substrate adaptor protein for the E3 ubiquitin ligase complex formed by CUL3 and RBX1 that targets Nrf2 for ubiquitination and degradation by the proteasome. During endogenous or exogenous stresses caused by either reactive oxygen species (ROS) or electrophilic chemicals, cysteine residues in Keap1 are modified, thereby inactivating its substrate adaptor function and disrupting the cycle of Nrf2 degradation.[21] This results in Nrf2 stabilization, its nuclear translocation, and the transcriptional upregulation of a multitude of antioxidant response element (ARE)-bearing genes that alleviate the stress response.[20]

Induction of Nrf2 signaling by thiol-reactive small molecules has demonstrated protective efficacy in chemoprevention tumor models and clinical trials.[22] As an example, sulforaphane (SFN), an aliphatic isothiocyanate with anti-inflammatory properties known to activate Nrf2,[23,24] has shown efficacy in men with high-grade prostatic intraepithelial neoplasia[25] and is being tested as a therapy for recurrent prostate cancer in phase II clinical trials.[26–28] Conversely, genetic analyses of human tumors have indicated that mutations and epigenetic modifications affecting the regulation of Nrf2 may cause resistance to chemotherapy through constitutive dominant hyperactivation of Nrf2 signaling.[29–31]

In this study, we demonstrate that the transcription factor Nrf2 is required to direct VSVΔ51 replication and oncolysis in some cancer cells. A combinatorial treatment of VSVΔ51 and the Nrf2 inducer SFN markedly increases viral replication and oncolysis in different cancer cell lines both in vitro and in vivo. We further show that Nrf2-constitutively active chemoresistant lung cancer (A549) cells are particularly vulnerable to VSVΔ51-driven oncolysis and do not require SFN treatment. Mechanistically, we show that either genetic or chemical induction of Nrf2 signaling suppressed the type I IFN response via increased autophagy. By transiently silencing *Nrf2* and *Atg7*, we demonstrate that Nrf2-mediated autophagy is critical for VSVΔ51 replication in chemoresistant A549 cells and bone osteosarcoma (U-2 OS) cells. Altogether, our findings highlight a previously unappreciated role for Nrf2 in the control of antiviral innate immunity and VSVΔ51 oncolysis.

RESULTS

SFN Enhances VSVΔ51 Replication and Oncolysis in PC-3 Cells

The capacity of SFN to augment VSVΔ51 replication was initially evaluated in human prostate cancer (PC-3) cells that display significant resistance to VSVΔ51-mediated cell killing.[10,12] SFN potentiated VSVΔ51-GFP infectivity in a dose-dependent manner, as measured by flow cytometry analysis of VSVΔ51-GFP$^+$ cells (Figures 1A and 1B). The number of VSVΔ51-infected cells increased from 4.2% with VSVΔ51 alone to 55.3% in combination with SFN (20 μM) at 48 hr (***$p < 0.001$) (Figure 1B). Similarly, the titer of VSVΔ51 released from infected PC-3 cells increased approximately 2 logs with SFN pretreatment (Figure 1C). In other human and murine cancer cell lines (DU145 and TS/A), the combination of SFN and VSVΔ51 also significantly increased infection when compared with VSVΔ51 alone (Figure S1).

To evaluate the cytotoxic effect of the VSVΔ51+SFN combination, PC-3 tumor cells were pretreated with increasing doses of SFN and subsequently infected with VSVΔ51 and analyzed for viability and apoptosis at various times post-infection (p.i.) by microscopy (Figure 1D) and flow cytometry (Figures 1E and 1F). Enhanced cell killing was elicited by the combinatorial treatment, but not by individual treatment, based on the proportion of annexin-V$^+$ cells and cell viability (Figure 1D; Figures S2A–S2C). The 24 hr point illustrates the number of infected GFP$^+$ cells and a low level of cell death; by 48 hr, the overall number of GFP$^+$ cells remained similar, but virus-induced cell death was readily detected (Figures 1B–1F). The VSVΔ51+SFN combination significantly enhanced VSVΔ51-mediated PC-3 cell killing (17.2% annexin-V$^+$ cells in VSVΔ51 versus 78.33% in VSVΔ51+SFN) (***$p < 0.001$) and decreased cell viability (80.7% in VSVΔ51 versus 17.2% in VSVΔ51+SFN) (***$p < 0.001$) at 72 hr p.i. (Figures 1E and 1F). A strong statistical correlation between percentage of annexin-V$^+$ cells at 48 hr p.i. and VSVΔ51 infectivity at 24 hr was confirmed by the nonparametric Spearman test ($r = 0.7774$; $p < 0.0001$; $n = 33$) (Figure 1G). In contrast, SFN only slightly increased VSVΔ51 infectivity and did not affect VSVΔ51 oncolysis in human non-transformed MRC-5 fibroblasts compared to PC-3 cells (Figures S3A and S3B).

To assess the involvement of the mitochondrial pathway in mediating VSVΔ51-induced cell death, PC-3 cells pretreated with SFN and infected with VSVΔ51 were stained with MitoSOX, a probe specific for mitochondria-derived ROS. The levels of mitochondrial ROS release were statistically associated with the number of annexin-V$^+$ cells at 48 hr p.i. ($r = 0.924$; $p < 0.0001$; $n = 36$) (Figure 1H). Furthermore, and consistent with the release of ROS at the mitochondria, downstream caspases—caspase-3 and caspase-7—were activated with the combinatorial treatment, leading to poly (ADP-ribose) polymerase (PARP) cleavage (Figure 1I) and indicating that SFN enhanced VSVΔ51 replication, which preceded mitochondrial-dependent apoptosis in PC-3 cells.

SFN-VSVΔ51 Combination Therapy Delays Tumor Progression and Improves Survival

The therapeutic effect of combined VSVΔ51+SFN treatment was next evaluated in vivo using both syngeneic and xenograft mouse tumor

cells at 24 hr and the percentage of apoptotic annexin-V$^+$ cells at 48 hr was calculated in PC-3 cells using a Spearman test ($n = 33$). (H) The same statistical test was used to calculate the correlation between the percentage of apoptotic annexin-V$^+$ cells and the percentage of MitoSOX$^+$ cells at 48 hr after infection ($n = 36$). (I) PC-3 cells were pretreated for 24 hr with SFN (20 μM) and challenged with VSVΔ51-GFP (MOI 1) for 48 hr. Whole-cell extracts (WCEs) were analyzed by immunoblotting for cleaved caspase-3, cleaved caspase-7 (high and low exposure), cleaved PARP, VSVΔ51-GFP, and β-actin. Results are from a representative experiment; all immunoblots are from the same samples.

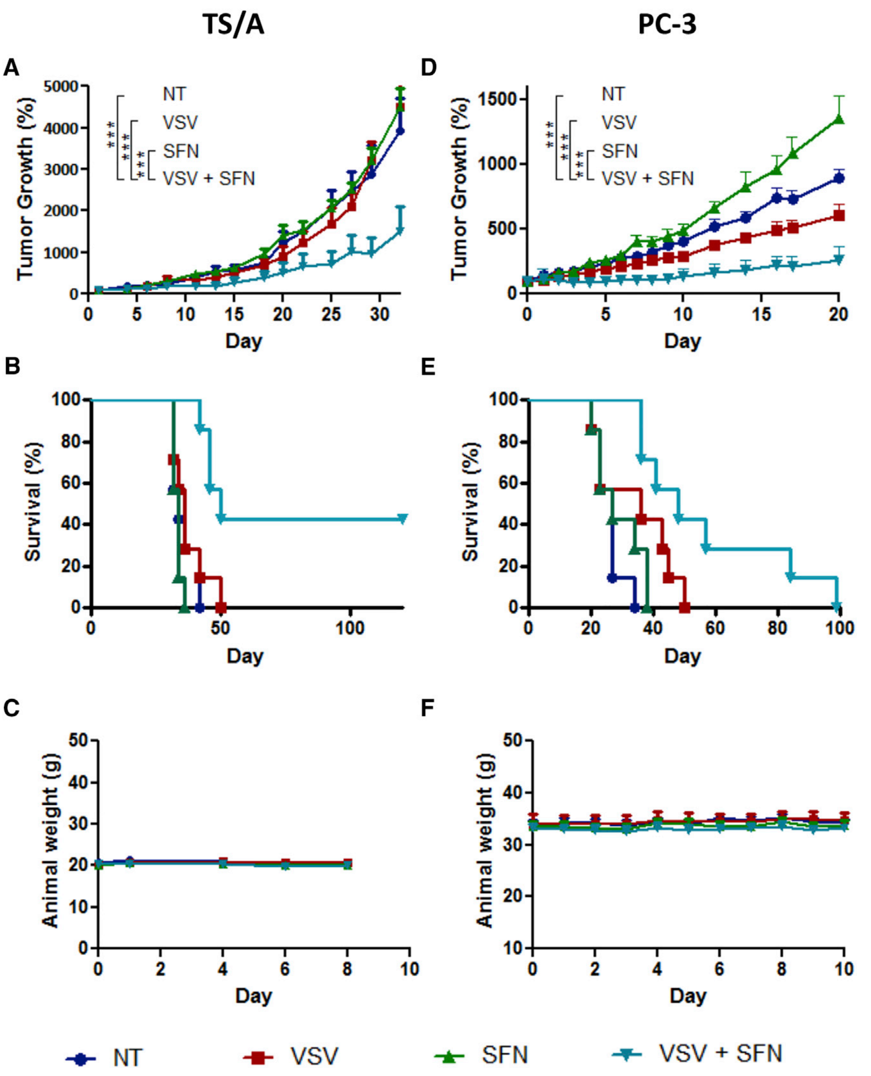

Figure 2. VSVΔ51 and Sulforaphane Combinatorial Treatment Reduces Tumor Progression and Prolongs Survival in Mouse Syngeneic and Xenograft Tumor Models

(A–C) A murine TS/A (3 × 10^5 cells) subcutaneous model was established in immunocompetent BALB/c mice (n = 7). After 7 days, when tumors became palpable, they were injected with 2 × 10^7 PFU of the VSVΔ51-GFP at day 0 and day 4. Sulforaphane (SFN) at 10 mg/kg was administered i.p. 1 day before the first VSVΔ51 injection and then days 1, 4, and 6 following the first VSVΔ51 administration. (A) Tumor growth was monitored using caliper measurements at the indicated times, and average tumor volumes (n = 7) are shown. To account for variable tumor size at the beginning of the experiment, tumor volume was normalized to its initial volume on day 0 and presented as percentage. Error bars correspond to the SEM. ***$p < 0.001$ by two-way ANOVA. (B) Cumulative survival rate (n = 7) was monitored over time. The log rank test indicates that the combined treatment is significantly prolonged over virus alone (**p = 0.0064) or SFN alone (***p = 0.0002). (C) The cytotoxicity of each treatment was assessed by monitoring mouse body weight. Error bars correspond to the SEM (n = 7). (D–F) Human PC-3 cells were established in athymic male nude mice. Four weeks later, animals were treated by intratumoral injection with VSVΔ51 (2 × 10^7 PFU/dose) on days 0, 3, 6, 9, and 12 and then treated intraperitoneally with SFN (10 mg/kg) every day from day −1 to day 10. (D) Tumor volume was monitored for each group, and average tumor volumes were normalized as shown (n = 7). To account for variable tumor size at the beginning of the experiment, tumor volume was normalized to its initial volume on day 0 and presented as percentage. Error bars correspond to the SEM. ***$p < 0.001$ by two-way ANOVA. (E) Survival rate (n = 7) was monitored over time. The log rank test indicates that the combined treatment is significantly prolonged over non-treated (***p = 0.0005) and SFN alone (**p = 0.0036). (F) The cytotoxicity of each treatment was assessed by monitoring mouse body weight (n = 7).

models. First, using a syngeneic immunocompetent mice tumor model engrafted subcutaneously (s.c.) with the murine breast cancer cell line TS/A, we demonstrate that murine breast tumor allografts were also significantly sensitized to intratumoral delivery of oncolytic VSVΔ51 (2 × 10^7 plaque-forming units [PFU]/injection) following intraperitoneal (i.p.) administration of SFN (10 mg/kg). Tumors treated with VSVΔ51+SFN displayed a 4-fold decrease in tumor volume, compared with animals receiving monotherapies (***$p < 0.001$ between VSVΔ51 and VSVΔ51+SFN) (Figure 2A); furthermore, combination treatment had a significant effect in prolonging survival (median survival in days: not treated [NT] = 34; SFN = 34; VSVΔ51 = 36; VSVΔ51+SFN = 50). The log rank test indicates that the combined treatment significantly prolonged survival over virus alone (**p = 0.0064) or SFN alone (***p = 0.0002). Interestingly, 43% (3 of 7) of the mice were cured as a result of VSVΔ51+SFN treatment (Figure 2B), without significant weight loss (Figure 2C). In athymic nude mice engrafted s.c. with human PC-3 cells, a delay in tumor progression (***$p < 0.001$ between VSVΔ51 and VSVΔ51+SFN) and prolonged survival was also observed with co-administration of VSVΔ51+SFN (Figures 2D and 2E), without weight loss (Figure 2F) (median survival in days: NT = 27; SFN = 27; VSVΔ51 = 36; VSVΔ51+SFN = 48). SFN treatment alone appeared to enhance tumor growth, in agreement with the cytoprotective role of Nrf2 activation. The log rank test also indicates that the combined treatment significantly prolonged survival over no treatment (***p = 0.0005) and SFN-alone treatment (**p = 0.0036) and is approaching statistical significance over virus-alone treatment (p = 0.0565). Collectively, these data demonstrate that the VSVΔ51+SFN combination in vivo is significantly more effective than monotherapy.

VSVΔ51 Relies on Nrf2 to Replicate and Drive Oncolysis

We next sought to investigate the mechanisms of SFN-mediated enhancement of VSVΔ51 replication in PC-3 cells by examining the activation of Nrf2 following SFN exposure by Phosflow

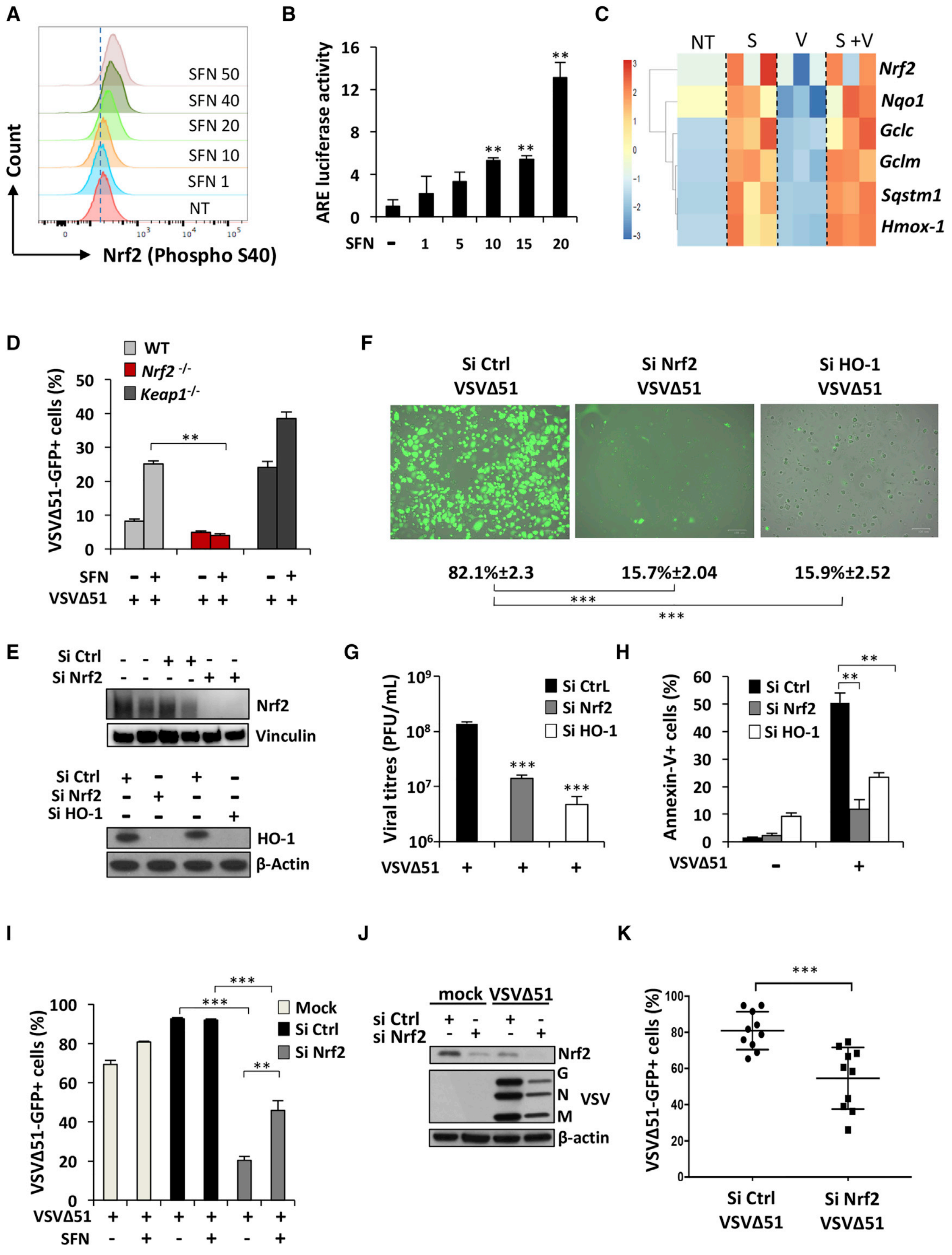

Figure 3. VSVΔ51 Replication Relies on Nrf2 and HO-1

(A) Intracellular levels of phosphorylated Nrf2 were detected by Phosflow in HEK293T stimulated for 18 hr with increasing doses of SFN. (B) HEK293T cells were pretreated for 24 hr with increasing doses of SFN, and the ARE promoter activity was assessed using a luciferase assay. (C) High-throughput analysis of gene expression was evaluated by qPCR BioMark analysis on PC-3 cells pretreated with SFN (20 μM) for 24 hr and subsequently infected with VSVΔ51-GFP (MOI 1) for 24 hr. Gene expression levels were calculated using the ΔΔCt method, and the gene-wise standardized expression (*Z* score) was generated for each gene. The scale represents *Z* score values, with red

(legend continued on next page)

cytometry. Increased levels of phosphorylated Nrf2 were observed as the dose of SFN increased (Figure 3A), and using an ARE promoter-luciferase assay, enhanced ARE promoter activity was observed with increasing SFN concentration (Figure 3B). Using high-throughput qRT-PCR analysis to examine the Nrf2-dependent gene expression profile in PC-3 cells, we observed the induction of a cross section of Nrf2-regulated genes following SFN treatment, independent of VSVΔ51 infection (Figure 3C). *Hmox-1* was the most highly induced Nrf2-stimulated gene after SFN treatment, as shown by an ~3-fold increase in mRNA expression level in both the presence and absence of VSVΔ51 (***$p < 0.001$) (Figure 3C). Another known inducer of Nrf2, diethyl maleate (DEM), increased ARE promoter activity and enhanced VSVΔ51 infectivity in a dose-dependent manner, with a ~4-fold increase in ARE activity at 100 μM (***$p < 0.001$) (Figure S4A); as with SFN, DEM enhanced VSVΔ51 infectivity in resistant PC-3 cells, as measured by flow cytometry analysis of VSVΔ51-GFP$^+$ cells (Figure S4B).

SFN was previously shown to stimulate Nrf2 partly through intracellular ROS accumulation.[32,33] To determine whether Nrf2 induction and VSVΔ51 replication were dependent on increased ROS release following SFN treatment, an oxidant-sensitive fluorescent detection probe, CM-H2DCFDA, was used to monitor ROS formation by flow cytometry. H_2O_2 and pyocyanin were used as positive controls to validate the oxidation of the probe and the detection of ROS accumulation. Increased ROS production was not observed at 4 hr or at 24 hr after PC-3 cell stimulation with SFN (Figure S5A). In addition, pretreatment of cells with the ROS scavenger N-acetyl-L-cysteine (L-NAC) did not interfere with the SFN-enhanced ARE-Luc promotor activity or VSVΔ51 infectivity, while L-NAC was effective at reducing ROS accumulation following treatment with pyocyanin, a potent ROS inducer (Figures S5B–S5D). These data indicate that induction of Nrf2 using electrophilic chemical agents potentiates VSVΔ51-infectivity independently of intracellular ROS accumulation in PC-3 cells.

To determine whether SFN-induced VSVΔ51 replication required the activation of Nrf2 and the downstream antioxidant response, we examined VSVΔ51 replication in murine embryonic fibroblasts (MEFs) deficient for Nrf2 (*Nrf2*$^{-/-}$), infected with VSVΔ51 in the presence or absence of SFN. Cells lacking Nrf2 had significantly lower infectivity rates when compared to wild-type (WT) MEFs after SFN treatment (**$p < 0.01$) (Figure 3D). To validate that SFN-induced VSVΔ51 infectivity relies on Nrf2, MEFs deficient for Keap1 (*Keap1*$^{-/-}$) were also examined. VSVΔ51 infectivity in *Keap1*$^{-/-}$ MEFs had similar levels of infection to WT MEFs treated with SFN (Figure 3D).[34] However, VSVΔ51 infectivity in *Keap1*$^{-/-}$ MEFs was enhanced by SFN treatment, suggesting that SFN has additional effects that are independent of the Nrf2/Keap1 axis.

The human A549 cell line possesses a point mutation in Keap1 that abolishes its capacity to repress Nrf2, leading to constitutive Nrf2-mediated gene expression.[31] Heme oxygenase-1 (HO-1) is one of the well-characterized Nrf2-regulated genes shown to modulate subcellular distribution and activation of Nrf2 to enhance antioxidant defenses.[35] To evaluate the importance of this axis in VSVΔ51 replication, chemoresistant A549 cells were transiently knocked down for Nrf2 or HO-1 using a small-interfering RNA (siRNA) approach (Figure 3E). A549 cells endogenously express high levels of HO-1 and were highly susceptible to VSVΔ51 infection (~80% infection with VSVΔ51 at MOI 0.01) (Figures 3E and 3F). A549 cells knocked down for Nrf2 or HO-1 had nearly 4-fold fewer VSVΔ51-GFP$^+$ cells (***$p < 0.001$) (Figure 3F) and a more than 10-fold decrease in virus titers compared to the siRNA control-treated cells (***$p < 0.001$) (Figure 3G). Nrf2 or HO-1 knockdown significantly reduced VSVΔ51-mediated cell killing, as shown by a ~2 to 3-fold decrease in the number of annexin-V$^+$ apoptotic cells (Figure 3H). The knockdown of Nrf2 also significantly affected the levels of VSVΔ51 infection in both the presence and the absence of SFN in A549 cells (Figure 3I). However, as observed in the *Keap1*$^{-/-}$ MEF cells, VSVΔ51 infectivity was slightly enhanced by SFN treatment, suggesting an Nrf2-independent effect of SFN in cells constitutively active for Nrf2. Finally, to broaden our observation that Nrf2 facilitates VSVΔ51 replication in cancer cells, U-2 OS cells were transiently knockdown for Nrf2 using an siRNA approach (Figure 3J). The lack of Nrf2 facilitated VSVΔ51 infection in U-2 OS cells, as demonstrated by a significant decrease in viral protein expression and infectivity (***$p < 0.001$) (Figures 3J and 3K). Collectively, these experiments indicate that VSVΔ51 relies on Nrf2 to replicate and drive oncolysis in A549 cells and U-2 OS cells.

VSVΔ51 Replication Is Dependent on Nrf2-Mediated Autophagy

Previous studies demonstrated that SFN can trigger autophagy in cancer cells[36] and that autophagy increases VSVΔ51 replication.[12,37] The ability of SFN to induce autophagy was examined by evaluating the levels of two autophagy biomarkers: the microtubule-associated

showing upregulation and blue showing downregulation in gene expression. Data are representative of three independent experiments. Each box of the heatmap represents one experiment. (D) WT, *Nrf2*$^{-/-}$, and *Keap1*$^{-/-}$ MEF cells were pretreated for 18 hr with SFN (10 μM) and subsequently challenged with VSVΔ51-GFP (MOI 0.1). Infectivity was quantified by flow cytometry 24 hr post-infection. Data are the means ± SEM of one representative experiment performed in triplicate. The experiment was repeated twice with the same trend. (E–H) A549 cells were transfected with control, Nrf2, or HO-1 siRNA and 48 hr later were infected with VSVΔ51-GFP (MOI 0.01) for an extra 24 or 48 hr. (E) Knockdown efficiency was assessed by immunoblotting. Viral infectivity and replication were determined by microscopy (F) and plaque assay (G) at 24 hr post-infection, respectively. (H) Apoptosis was determined by flow cytometry 48 hr post-infection. Data are the means ± SEM of one representative experiment performed in triplicate. Experiment was repeated twice with the same trend. (I) A549 cells were transfected with control or Nrf2 siRNA and 48 hr later were infected with VSVΔ51-GFP (MOI 0.01) in the presence or absence of SFN (7.5 μM) for an extra 24 hr. Viral infectivity was determined by flow cytometry. Data are the means ± SEM of two independent experiments performed in duplicate. (J) U-2 OS cells were transfected with control or Nrf2 siRNA and 48 hr later were infected with VSVΔ51-GFP (MOI 0.001). Whole-cell extracts were also analyzed by immunoblotting for Nrf2, VSV proteins, and β-actin. (K) Viral infectivity was determined by flow cytometry 24 hr post-infection. Data are the means ± SEM of three independent experiments.

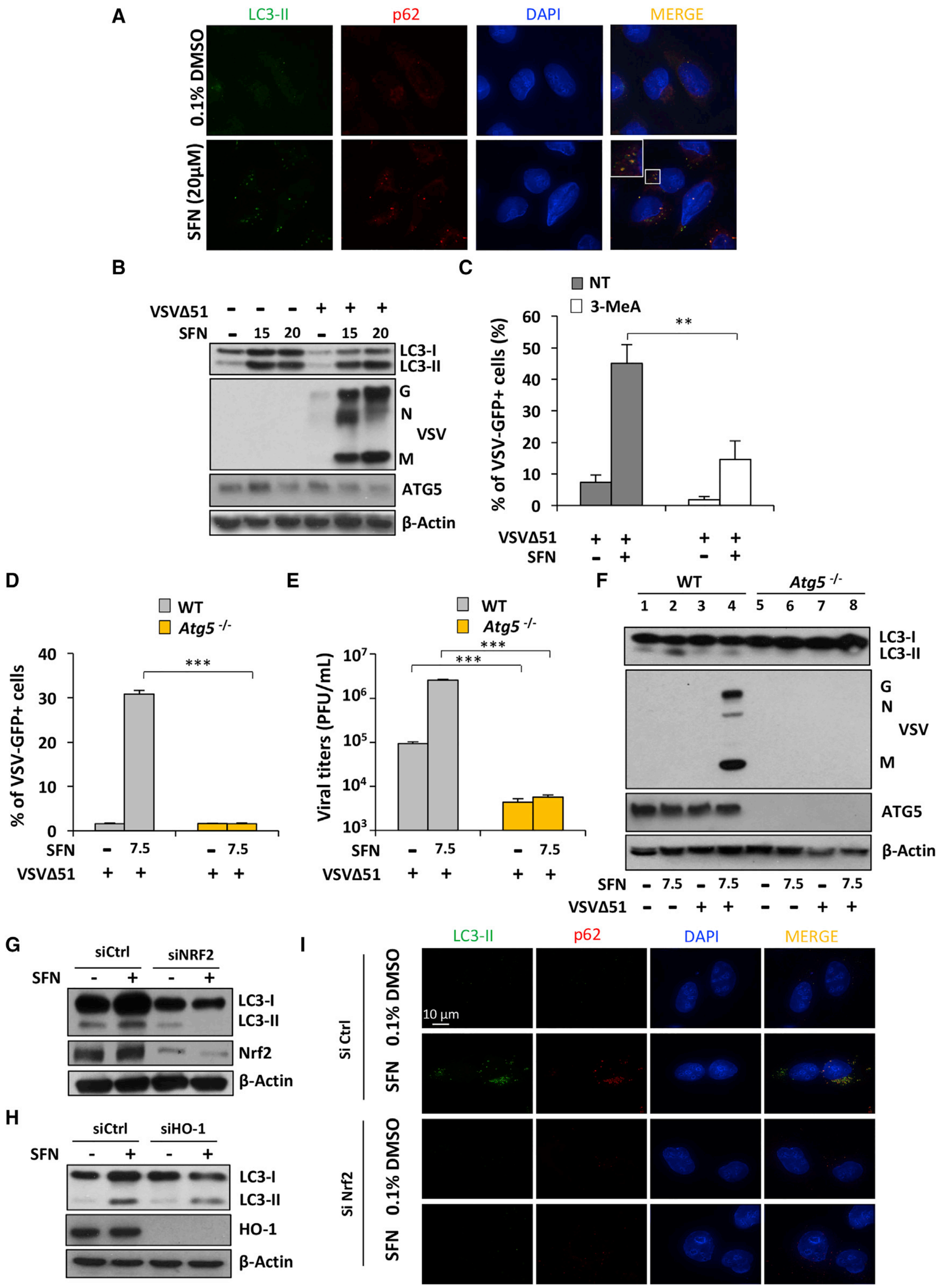

Figure 4. Sulforaphane-Enhanced VSVΔ51 Replication and Oncolysis Is Dependent on Nrf2-Dependent Autophagy

(A) Immunofluorescence analysis of U-2 OS cells was conducted to detect changes in the levels of p62 and LC3-II in response to a 24 hr treatment with SFN (20 μM). (B) PC-3 cells were pretreated with SFN (20 μM) for 24 hr and were subsequently infected with VSVΔ51-GFP (MOI 1). Whole-cell extracts were analyzed by immunoblotting for LC3B,

(legend continued on next page)

light chain 3 protein (LC3) isoform II and the autophagic cargo protein p62/SQSTM1. Immunofluorescence showed increased levels of both p62 and LC3-II and further revealed that these two proteins co-localize in the cytoplasm of SFN-treated U-2 OS cells (Figure 4A). The same was observed in PC-3 cells; a dose optimization established that exposure to SFN caused an increase in the lipidated form of LC3-II (Figure 4B), and PC-3 cells treated with the VSVΔ51+SFN combination generated more lipidated LC3-II than cells infected with VSVΔ51 alone (Figure 4B). Furthermore, when 3-methyladenine (3-MeA), a general phosphatidylinositol 3-kinase (PI3K) and autophagy inhibitor, was used in combination with SFN, a 3-fold reduction in the number of infected cells was observed (**$p < 0.01$) (Figure 4C), suggesting the possible involvement of autophagy in driving SFN-increased VSVΔ51 infectivity in PC-3 cells.

To unequivocally demonstrate the involvement of autophagy in SFN-mediated VSVΔ51 replication and infectivity, MEFs deficient for Atg5, an essential autophagic protein responsible for autophagosomal elongation, were used. *Atg5*$^{-/-}$ MEFs showed no significant increase in VSVΔ51-GFP^{+} cells or virus titers when treated with SFN, whereas the WT MEFs had approximately a 10-fold increase in the number of VSVΔ51-GFP^{+} cells and ~50-fold higher virus titers compared to *Atg5*$^{-/-}$ cells (Figures 4D and 4E). A similar increase in LC3-II accumulation was observed in WT MEFs with the VSVΔ51+SFN combination. In contrast, *Atg5*$^{-/-}$ MEFs showed no VSVΔ51 protein expression and a marked reduction in increased levels of LC3-II (Figure 4F), indicating that SFN-stimulated autophagy facilitates VSVΔ51 replication.

Because both Nrf2 activation and autophagy depend on stress induction, we investigated whether Nrf2 activation regulates autophagy. Using siRNA-mediated silencing, Nrf2 expression was knocked down in A549 cells, leading to abrogation of LC3-II expression upon SFN treatment and suggesting that autophagy is partly dependent on Nrf2 expression in these cells (Figure 4G). These results agree with a report showing that Nrf2 regulates the gene expression of a number of proteins involved in the autophagy process.[38] The same was observed upon HO-1 knockdown in A549 cells, which suppressed SFN-induced increase in LC3-II (Figure 4H). The result was also observed in siRNA Nrf2 (siNrf2)-transfected U-2 OS cells, because no increase in LC3-II or p62 was detected in the absence of Nrf2 following SFN stimulation (Figure 4I). Collectively, these results demonstrate that VSVΔ51 replication is dependent on Nrf2/HO-1-mediated autophagy in different cancer cell lines.

SFN Dampens the Innate Antiviral Response

To investigate whether SFN modulates the antiviral response, HEK293T cells were transfected with the IFN-stimulated response element (ISRE)-luciferase reporter plasmid (Luc) and infected with VSVΔ51 in the presence or absence of increasing doses of SFN. SFN inhibited VSVΔ51-mediated induction of ISRE-Luc activity in a dose-dependent manner (Figure 5A). Using a high-throughput qPCR approach, we assessed the levels of IFN-stimulated genes (ISGs), including *Ccl5*, *Cxcl10*, *Tmem173*, *Ddx58*, and *Irf7*, which were all highly induced by VSV infection, whereas SFN treatment inhibited ISG expression; VSVΔ51-induced *Ifnb1* and *Il-29* mRNA were drastically inhibited by SFN by more than 50 and 500 times, respectively (Figure 5B). Other ISGs, such as *Ifitm1*, *Rsad2*, *Mx1*, *Isg15*, *Isg56*, and *Oasl*, were also inhibited in SFN-treated cells (Figure 5B). Phosphorylation of IRF3 and STAT1 and the subsequent expression of ISGs such as STAT1 and STING were drastically inhibited at the protein level by the presence of SFN (Figure 5C). The decreased antiviral response also correlated with induced expression of HO-1 (Figure 5C), illustrating that increased VSVΔ51 infection correlates with an Nrf2-mediated inhibition of the host antiviral response. IRF3 is a critical transcription factor required for type I IFNs and ISG expression;[39] because IRF3 phosphorylation was affected by SFN treatment, we examined whether SFN also prevented IRF3 nuclear translocation and binding activity following VSVΔ51 infection. SFN interfered with IRF3 nuclear relocation, as denoted by the absence of IRF3 in nuclei of VSVΔ51+SFN-stimulated cells (Figure 5D). Nuclear translocation of Nrf2 was also observed in the same conditions (Figure 5D). As a result of IRF3 nuclear translocation impairment in SFN-treated cells, IRF3 failed to bind to its cognate binding site following SFN stimulation (Figure S6).

Nrf2-Mediated Autophagy Dampens VSVΔ51-Induced Innate Antiviral Response

Using Sendai virus as a potent stimulator of the antiviral response, Nrf2 overexpression inhibited virus-mediated ISRE-Luc activity in a dose-dependent manner (Figure 6A), and knockdown of Nrf2 in A549 cells led to higher levels of P-STAT1, STAT1, and RIG-I in response to VSVΔ51 (Figure 6B). Increased expression of these antiviral markers correlated with a profound inhibition of the autophagic flux and a decreased capacity for VSVΔ51 to replicate in A549 cells silenced for Nrf2 (Figure 6B).

Our data have shown that VSVΔ51 replication also relies on the Nrf2-regulated gene HO-1. Using the observation that upregulation of autophagy increases VSVΔ51 replication, we investigated whether

VSV proteins, ATG5, and β-actin. Results are from a representative experiment; all immunoblots are from the same samples. (C) PC-3 cells were pretreated with SFN (20 μM) in the presence or absence of the PI3K/autophagy inhibitor 3-MeA (10 mM) for 24 hr. Cells were subsequently infected with VSVΔ51-GFP (MOI 1). Infectivity was quantified by flow cytometry at 24 hr post-infection. Data are the means ± SEM from three independent experiments. (D–F) WT *Atg5* and *Atg5*$^{-/-}$ MEFs were stimulated overnight with SFN (7.5 μM) before challenge with VSVΔ51 (MOI 0.01). Viral infectivity was determined by flow cytometry (D) and viral replication by plaque assay (E). (F) Whole-cell extracts were analyzed by immunoblotting for LC3B, VSV proteins, ATG5, and β-actin. (G and H) A549 cells were transfected with control, Nrf2, or HO-1 siRNA and 48 hr later were treated with SFN (15 μM) for an extra 24 hr. Whole-cell extracts were analyzed by immunoblotting for Nrf2, HO-1, LC3, and β-actin. (I) Immunofluorescence analysis of U-2 OS cells was conducted to detect the changes in the levels of p62 and LC3-II in response to a 24 hr treatment with SFN (20 μM) in the presence of Nrf2 (siCtrl) or absence of Nrf2 (siNrf2).

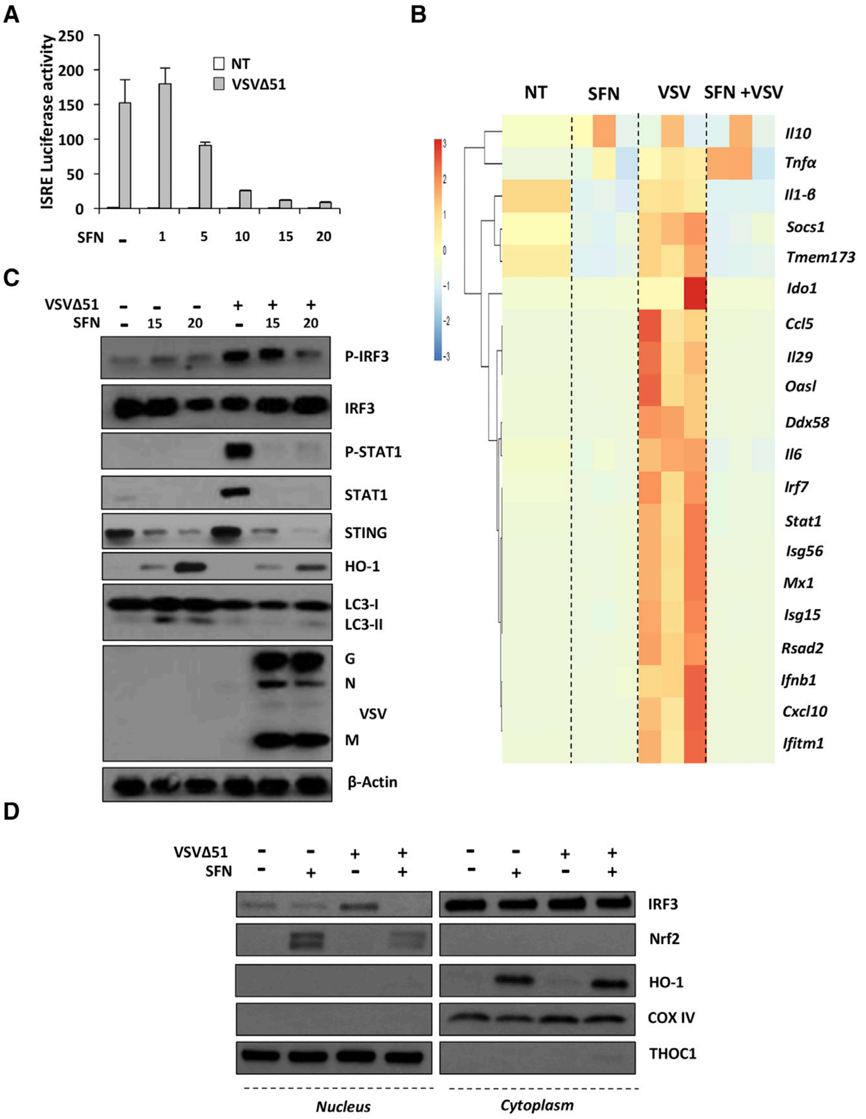

Figure 5. Sulforaphane Dampens the Innate Antiviral Response

(A) HEK293T cells were pretreated for 24 hr with increasing doses of SFN (1–20 μM), and the ISRE promoter activity was assessed using a luciferase assay in the presence or absence of VSVΔ51 (MOI 0.01). (B) PC-3 cells were pretreated with sulforaphane (SFN) (20 μM) for 24 hr and were subsequently infected with VSVΔ51-GFP (MOI 1). Antiviral and inflammatory gene expression levels were assessed by high-throughput qPCR. (C) PC-3 cells were pretreated for 24 hr with SFN (20 μM) and subsequently challenged with VSVΔ51-GFP (MOI 1) for 24 hr. Whole-cell extracts were analyzed for P-IRF3, IRF3, P-STAT1, STAT1, STING, HO-1, LC3, VSV proteins, and β-actin by immunoblotting. (D) PC-3 cells were pretreated for 24 hr with SFN (20 μM) and subsequently challenged with VSVΔ51-GFP (MOI 1) for 8 hr. Translocation of IRF3 and Nrf2 from cytoplasm to nucleus was investigated by immunoblotting in cytoplasmic and nuclear fractions at the same time. The experiment was repeated twice.

the dampening of the antiviral response was also related to HO-1-mediated autophagy. Increased expression of HO-1 in HEK293T cells inhibited Sendai virus-mediated ISRE activity in a dose-dependent manner (Figure 6C). Similar to the observations in Nrf2-silenced cells, HO-1 knockdown resulted in higher levels of the antiviral effectors P-STAT1, STAT1, and RIG-I following VSVΔ51 infection, compared to HO-1-sufficient cells (Figure 6D). Stimulation of the antiviral response correlated with decreased VSVΔ51 replication and expression of the autophagy marker LC3-II (Figure 6D). Altogether, genetic inhibition of both Nrf2 and HO-1 limited autophagy in A549 cells and led to increased antiviral immunity, which further limited VSVΔ51 replication.

Previously, Jounai et al.[37] demonstrated, using *Atg5* and *Atg7* knockout (KO) MEFs, that autophagy increased virus replication by suppressing the innate antiviral response. To validate this relationship, Atg7 expression was silenced in A549 cells; cells knocked down for Atg7 had 2-fold fewer VSVΔ51-GFP^{+} cells (***$p < 0.001$) (Figure 6E; Figure S7A) and an ~1 log decrease in virus titers compared to the siRNA control-treated cells (*$p < 0.05$) (Figure S7B). Atg7 knockdown resulted in a marked increase in P-STAT1 and RIG-I expression that correlated with >90% inhibition of VSVΔ51 protein expression (Figure 6F), thus demonstrating that inhibition of autophagy also increased the antiviral response in A549 cells.

To further evaluate the effect of autophagy on VSVΔ51 replication and antiviral responses, U-2 OS cells were silenced for Nrf2 using siRNA. Cells lacking Nrf2 had reduced VSVΔ51 infectivity and replication (Figures 6G and 6H), which correlated with a reduction in LC3-II expression and increased STAT1 signaling, as denoted by the elevated level of STAT1 phosphorylation (Figure 6H). Collectively, our data demonstrate that induction of Nrf2 signaling drives autophagy to decrease innate antiviral responses and potentiate VSVΔ51 replication and oncolytic activity in different cancer cell models (Figure 7).

DISCUSSION

Many tumor cell lines and primary cancer specimens exhibit resistance to infection by OVs, partly because of persistent antiviral immunity.[6] In the effort to increase the efficacy of experimental oncolytic virotherapies, we and others have explored strategies to potentiate viral oncolysis in resistant cancer cells by combining OVs with small molecules that enhance virus replication and cell killing.[7–14] In the present study, we demonstrate that the restriction

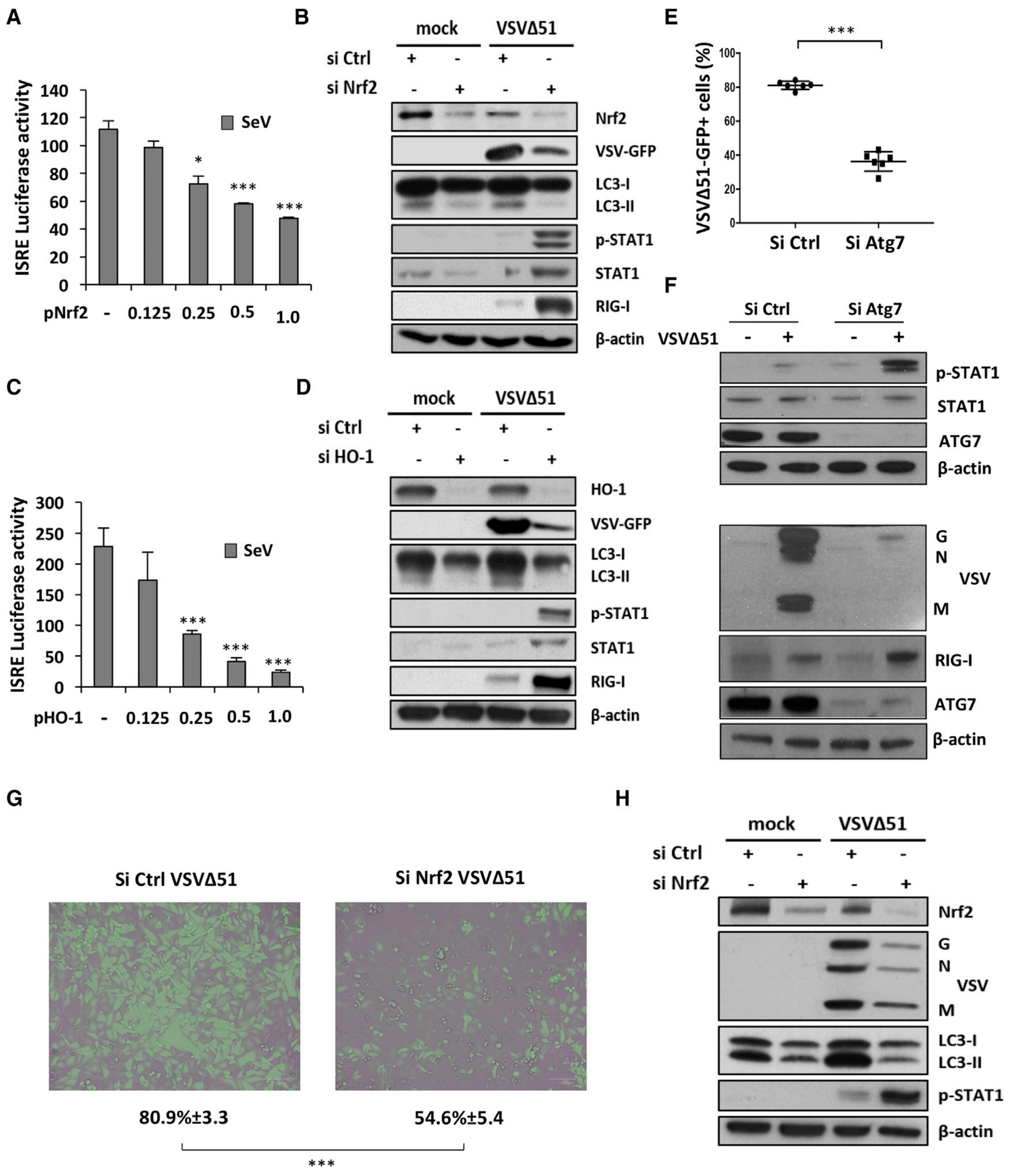

Figure 6. Nrf2-Induced Autophagy Curtails Antiviral Innate Immunity

(A) 6 hr following transfection with increasing doses of an Nrf2 overexpression plasmid, HEK293T cells were infected with Sendai virus (SeV) (40 hemagglutinin [HA]/mL) for 18 hr and ISRE promoter activity was assessed by a luciferase reporter assay. (B) A549 cells were transfected with control or Nrf2 siRNA and 48 hr later were challenged with VSVΔ51-GFP (MOI 0.01) for an extra day. Whole-cell extracts were analyzed for Nrf2, VSV-GFP, LC3, P-STAT1, STAT1, RIG-I, and β-actin by immunoblotting. (C) 6 hr following transfection with increasing doses of an HO-1 overexpression plasmid, HEK293T cells were infected with SeV (40 HA/mL) for 18 hr and ISRE promoter activity was assessed by a luciferase reporter assay. (D) A549 cells were transfected with control or HO-1 siRNA and 48 hr later were challenged with VSVΔ51-GFP (MOI 0.01) for an extra day. Whole-cell extracts were analyzed for HO-1, VSV-GFP, LC3, P-STAT1, STAT1, RIG-I, and β-actin by immunoblotting. (E and F) A549 cells were transfected with control or Atg7 siRNA and 48 hr later were challenged with VSVΔ51-GFP (MOI 0.01) for an extra day. (E) VSVΔ51 infection was determined by flow cytometry. Data are the means ± SEM of three independent experiments. (F) Whole-cell extracts were analyzed for VSV proteins, RIG-I, P-STAT1, STAT1, and β-actin by immunoblotting. (G and H) U-2 OS cells were transfected with control or Nrf2 siRNA and 48 hr later were infected with VSVΔ51-GFP (MOI 0.001). (G) Viral infectivity was determined by flow cytometry 24 hr post-infection and observed using a fluorescent microscope. Data are the means ± SEM of three independent experiments. (H) Whole-cell extracts were also analyzed by immunoblotting for Nrf2, VSV proteins, LC3, P-STAT1, and β-actin.

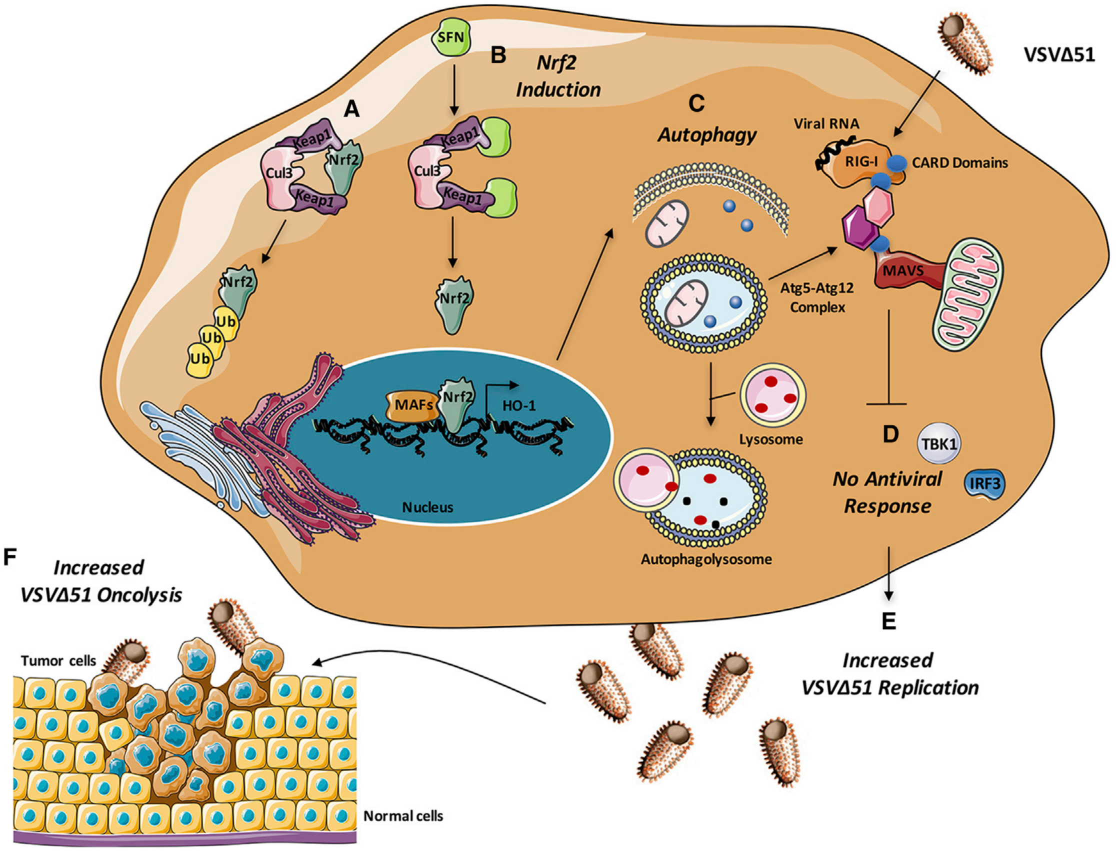

Figure 7. Nrf2-Dependent Autophagy Decreases the Innate Antiviral Response to Augment VSVΔ51 Replication and Oncolytic Activity in Cancer Cells
(A) Under homeostatic conditions, Nrf2 is sequestered in the cytoplasm by Keap1, a substrate adaptor protein for a E3 ubiquitin ligase complex that targets Nrf2 for ubiquitination and degradation by the proteasome. (B) Upon stimulation with sulforaphane (SFN), cysteine residues in Keap1 are modified, thereby inactivating its substrate adaptor function and disrupting the cycle of Nrf2 degradation. This results in Nrf2 stabilization, its nuclear translocation, and the transcriptional upregulation of a multitude of genes bearing antioxidant response element (ARE) in their promoter, including HO-1. (C and D) Increased Nrf2 signaling induces autophagy (C), which negatively regulates VSVΔ51-induced immune response (D) by disrupting RIG-I-MAVS interactions. (E and F) Suppression of the antiviral response facilitates VSVΔ51 replication (E) and oncolysis (F) in cancer cells.

to VSVΔ51 oncolysis can be overcome by manipulation of the oxidative stress response using the antioxidant compound SFN as an inducer of Nrf2 antioxidant activity. We demonstrated that (1) SFN selectively increased VSVΔ51 infectivity and cell death in cancer cells, (2) the combination of SFN and VSVΔ51 decreased tumor volume and increased survival in vivo, (3) VSVΔ51 replication was dependent on activation of the Nrf2 pathway, (4) VSVΔ51 replication relied on Nrf2/HO-1-mediated autophagy, and (5) activation of autophagy through the Nrf2/HO-1 axis suppressed the innate antiviral response by inhibiting IRF3 activity.

SFN is the major isothiocyanate derived from broccoli,[40] produced by the enzymatic hydrolysis of glucosinolates that are present in cruciferous plants, including broccoli sprouts.[41] Glucoraphanin, the predominant glucosinolate, is biologically inactive but is transformed into SFN by the enzymatic activity of myrosinase. SFN is rapidly absorbed in the blood and possesses antioxidant, anti-inflammatory, and cytoprotective activities. The anticancer potential of SFN has been observed in cancer cells, in carcinogen-induced and genetic animal cancer models, and in xenograft models of cancer.[42,43] Furthermore, the combination of SFN with existing cancer chemotherapeutics was shown to increase the cytotoxic effect in vitro,[42] thus suggesting a potential therapeutic benefit in the clinical setting.

SFN treatment stimulated Nrf2 expression and dampened the antiviral innate immune response via upregulation of autophagy, thus facilitating OV-mediated cell killing both in vitro and in vivo. Mechanistically, SFN prevented IRF3 nuclear translocation and binding to its cognate binding element, thus preventing downstream STAT1 activation and the induction of ISGs. In A549 cells, the antiviral response to VSVΔ51 was suppressed because of the constitutive activation of Nrf2; antiviral immunity was stimulated only when Nrf2, HO-1, or Atg7 was selectively silenced using siRNA, thus reflecting the importance of the Nrf2 axis and the autophagy

pathway in regulating VSVΔ51-induced immune response. These observations are in agreement with Jounai et al.,[37] who demonstrated that the Atg5-Atg12 autophagy complex negatively regulated IRF3 signaling by preventing the association between RIG-I and MAVS.

Furthermore, Nrf2 has been shown to regulate the expression of a number of proteins involved in macroautophagy[38] as well as ATG3, an E2-like enzyme required for the conjugation of LC3B to phosphatidylethanolamine. In addition to its role as a transcriptional regulator of the antioxidant response pathway, Nrf2 positively regulated autophagy and is an essential component of regulatory networks that respond to different types of stress, including protein aggregation, nutrient deficiency, and viral infection. Although previously reported that SFN action is dependent on ROS production in PC-3 cells,[44] we demonstrated that SFN alone did not stimulate ROS accumulation and the effects of SFN on Nrf2 activity and VSVΔ51-induced infectivity were independent of ROS. Activation of Nrf2 by SFN was previously shown to be independent of ROS, because SFN directly binds to cysteine residues within the Nrf2 repressor Keap1 in vitro,[34,45] with C151 serving as the main sensor cysteine.[46,47]

The Nrf2-regulated gene HO-1 was one of the Nrf2-regulated activities responsible for the induction of autophagy. It is possible that HO-1 activates autophagy via the ataxia telangiectasia mutated (ATM) protein, which triggers autophagy by inhibiting mTORC1.[48] Nrf2 positively regulates ATM during cell stress, and ATM is activated by the accumulation of carbon monoxide, a product of HO-1 catalysis.[49] Thus, HO-1 may activate autophagy through the production of carbon monoxide, which then activates ATM, inhibits mTORC1, and facilitates autophagy. In support of this idea, blockade of type I IFN production by rapamycin inhibited mTORC1 and potentiated the oncolytic activity of VSVΔ51.[50] Consistent with the preceding idea, the mTOR pathway was inhibited following SFN treatment in PC-3 cells (D.O., unpublished data), and SFN was shown to induce autophagy through the inhibition of mechanistic target of rapamycin (mTOR) in primary murine hepatocytes.[51] Because inhibition of mTOR is crucial to the potentiation of VSVΔ51 oncolysis,[50] further studies are required to investigate the relationship between mTOR signaling and stimulation of VSVΔ51 replication by SFN.

In addition to suppression of the innate antiviral response, stimulation of the adaptive immune response by OV infection is an essential part of a curative response.[4,52–54] Cancer cells reside in the complex host-tumor microenvironment and have evolved to suppress signaling to the adaptive immune system to evade T cell-mediated detection and destruction.[3,4,54] By dampening the innate antiviral response using oncolytic adjuvants, VSVΔ51 and other OVs replicate more robustly within the tumor mass, increase inflammation in the tumor bed, and facilitate an adaptive T cell-mediated response against tumor antigens via cross presentation.[52,54–56] Although more detailed mechanistic studies are required to investigate the effects of SFN in vivo, we speculate that SFN enhances the therapeutic effect of VSVΔ51 in tumor models by a similar mechanism. We also speculate that the small tumor volumes associated with the TS/A model in BALB/c mice correlated with the immunocompetent environment that targeted the TS/A tumor cells, in contrast to the immunodeficient environment associated with the PC-3-nude mouse model. Our results, along with numerous other studies, demonstrate that combinatorial oncolytic virotherapy culminates in multiple mechanisms of tumor killing: direct virus-mediated cell death, stimulation of inflammation within the tumor, and induction of a targeted adaptive immune response.[2,54,57]

The cancer cell line most susceptible to VSVΔ51-mediated cell killing was the human lung epithelial A549 line that harbors a point mutation inactivating Keap1, thus increasing Nrf2 nuclear translocation and transcriptional activity.[31] High levels of Nrf2 correlate with poor prognosis, and often patients bearing tumors with high amounts of nuclear Nrf2 are resistant to chemotherapy and radiation therapy.[31] An OV approach could be advantageous in patients with chemo- and radio-resistant Nrf2-associated tumors, because OV activity may be preferentially increased in this subset of tumors. Our studies are being directed toward establishing correlations among Nrf2 activation, antiviral suppression, and susceptibility to OV replication in tumors bearing mutations of the Nrf2/Keap1 axis. With regard to the transition of this and related combinatorial strategies to the clinic, both OVs and SFN are being evaluated as monotherapies in clinical trials. SFN, at concentrations that are achievable in vivo, has been shown to be non-toxic to healthy cells,[26,58,59] and mice exhibited no visible adverse effects to SFN; likewise, OV administration is well tolerated in multiple clinical trials.[2,57] Although we demonstrated that the SFN-mediated inhibition of the antiviral responses and enhanced OV efficacy is tumor cell specific, investigation of this promising combination approach could facilitate clinical implementation of OVs as therapeutic agents for the treatment of a variety of cancer types.

Collectively, our data demonstrate that SFN stimulates an Nrf2-regulated, multi-functional, transcriptional network that, in the context of virus infection, leads to the induction of autophagy, suppression of the IFN-mediated antiviral response, and subsequent enhancement of VSVΔ51 replication and tumor cell death. Furthermore, our findings reveal a previously unappreciated role for Nrf2 in controlling VSVΔ51 replication that could ultimately provide to patients with Nrf2-associated tumors an alternative form of immune-based therapy.

MATERIALS AND METHODS

Cell Lines and Culture Conditions

Human PC-3 and A549 cell lines were obtained from the American Type Culture Collection (ATCC). PC-3 and A549 cells were cultured in F12K medium (Wisent) supplemented with 10% fetal bovine serum (FBS, Wisent) and 1% antibiotics (Sigma-Aldrich). HEK293T cells were also obtained from ATCC and cultured in DMEM (Wisent) supplemented with 10% FBS and 1% antibiotics. TS/A cells (murine mammary adenocarcinoma) cultured in DMEM

supplemented with 10% FBS and 1% antibiotics were a gift from Dr. Glen Barber (University of Miami). MDA-MB-231 (mammary adenocarcinoma) human cells and Vero cells were provided by Drs. Josie Ursini-Siegel and Mark Wainberg, respectively (Lady Davis Institute) and were grown in DMEM, 10% FBS, and 1% antibiotics. Human U-2 OS cells were obtained from Dr. Sonia Rocha (University of Dundee) and were cultured in DMEM (Gibco) that contains L-glutamine, high D-glucose (4.5 g/L), and phenol red supplemented with 10% (v/v) FBS (Thermo Scientific). MRC-5 cells (human normal lung fibroblast) were obtained from Dr. Jerry Pelletier (McGill University) and cultured in DMEM supplemented with 10% FBS and 1% antibiotics.

WT, *Keap1*-knockout (*Keap1*$^{-/-}$), and *Nrf2*-knockout (*Nrf2*$^{-/-}$) MEFs were obtained from Dr. Masayuki Yamamoto (Tohoku University). MEF cell lines were generated from day 13.5 embryos of WT, *Nrf2*$^{-/-}$, and *Keap1*$^{-/-}$ C57BL/6 mice[60,61] and were cultured as previously described.[62] WT *Atg5* (*Atg5*$^{+/+}$) and *Atg5*-knockout (*Atg5*$^{-/-}$) MEFs were a gift from Dr. Yu-Ying He (University of Chicago) and were originally produced by Dr. Noboru Mizushima.[63] MEF cells were cultured in DMEM supplemented with 10% FBS and 1% antibiotics. All cell lines were incubated at 37°C in a humidified atmosphere of 5% CO_2 and 95% air.

Drugs and Cytokine

SFN and DEM were purchased from Santa Cruz Biotechnology and dissolved in DMSO and 95% ethanol, respectively. 3-MeA, L-NAC, pyocyanin, and H_2O_2 were purchased from Sigma-Aldrich. The pH of the L-NAC solution was adjusted to 7. IFN-α2b was obtained from Merck.

VSV Production, Quantification, and Infection

VSVΔ51-expressing GFP is a recombinant derivative of the VSV Indiana serotype described previously.[18] The virus was provided by Drs. John Bell and David Stojdl (Ottawa Health Research Institute). VSVΔ51 was propagated on Vero cells, and virus titers were quantified by a standard plaque assay method on Vero cells as described previously.[9,10]

In Vitro Treatment with Drugs and Infection with VSVΔ51

Cancer cell lines or MEFs were pretreated for 24 hr with SFN. Subsequently, cells were infected at indicated MOIs with VSVΔ51 for the indicated periods. The cells were imaged with the ZOE Fluorescent Cell Imager (Bio-Rad). The percentage of cells infected with VSVΔ51 was determined based on GFP expression using a BD LSR Fortessa cell analyzer (Becton Dickinson); calculations, as well as population analyses, were done using FACSDiva.

Flow Cytometry

Cell Viability and Apoptosis Analyses

Cell viability and apoptosis were assessed as previously reported[64] by flow cytometry and were based on the measurement of phosphatidylserine externalization at the cell surface using a specific annexin-V antibody.

Total and Mitochondrial ROS Production

Total ROS and mitochondrial-dependent ROS using CM-H2DCFDA (1 μM) and MitoSOX Red Mitochondrial Superoxide Indicator (0.5 μM, Life Technologies) were evaluated by flow cytometry. Following SFN treatment and/or VSVΔ51 infection, cells were washed in PBS before incubation with the different probes for 30 min at 37°C. After incubation, cells were washed twice in PBS before fluorescence-activated cell sorting (FACS) analysis.

Nrf2 Phosflow

Phospho-Nrf2 staining was performed in a 96-well plate format. Cells were resuspended in 100 μL of PBS and fixed with the same volume of prewarmed Fix Buffer I (BD Biosciences) for 10 min at 37°C. Cells were pelleted down by centrifugation and resuspended in 200 μL ice-cold Perm Buffer III (BD Biosciences) for 20 min at 4°C. Cells were then washed three times with 200 μL of PBS containing 5% FBS and incubated for 30 min on ice in PBS containing 2% FBS. Cells were pelleted down by centrifugation and stained in 100 μL PBS containing 2% FBS with a rabbit monoclonal phospho-S40-Nrf2 antibody (ab76026, Abcam) for 30 min at room temperature (RT). Cells were washed twice in staining buffer and stained with an Alexa Fluor 488 (AF488)-conjugated goat antirabbit secondary antibody (ab150077, Abcam) for an extra 30 min at RT. After two more washes using the staining buffer, cells were analyzed by flow cytometry.

Protein Extraction and Immunoblot Analysis

Cells were washed twice with ice-cold PBS and lysed in ice-cold buffer containing 10% glycerol, 20 mM Tris-HCl (pH 7.5), 1% Triton X-100, 5 mM NaF, 40 mM β-glycerophosphate, 1 mM Na_3VO_4, 150 mM NaCl, 1 mM PMSF, 1 mM DTT, 10 mM NEM, and protease inhibitor cocktail (Sigma-Aldrich). Lysed extracts were kept on ice for 30 min and centrifuged at 10,000 × *g* for 15 min at 4°C. Then, 20–40 μg of clarified whole-cell lysates were separated by SDS-PAGE on 4%–12% precast gradient gels (Bio-Rad). Proteins were electrophoretically transferred to a nitrocellulose membrane (0.45 μM, Bio-Rad) for 1 hr at 100 V in a buffer containing 30 mM Tris, 200 mM glycine, and 20% ethanol. Blots were blocked for 1 hr with 5% nonfat dried milk in PBST (PBS + 0.1% Tween 20) and then probed overnight at 4°C with any of the following specific primary antibodies: VSV-whole virus antisera, anti-GFP (sc-9996, Santa Cruz), anticleaved caspase-3 (9661, Cell Signal), anti-caspase-7/cleaved caspase-7 (9494, Cell Signal), anti-PARP/cleaved PARP (9541, Cell Signal), anti-Nrf2 (ab62352, Abcam; 12721, Cell Signal), anti-HO-1 (5853, Cell Signal), anti-P-IRF3 (4947, Cell Signal), anti-IRF3 (ab68481, Abcam), anti-P-STAT1 (9167, Cell Signal), anti-STAT1 (sc-417, Santa Cruz), anti-RIG-I (Bleed, Millipore), anti-STING (13647, Cell Signal), anti-LC3B (2775, Cell Signal), anti-Atg7 (8558, Cell Signal), and anti-actin (MAB1501, Millipore) used as loading control. After three washes in PBST, antibody signals were detected by chemiluminescence using secondary mouse or rabbit antibodies conjugated to horseradish peroxidase (Mandel) and an enhanced chemiluminescence (ECL) detection kit as per manufacturer's instructions (Thermo Scientific).

RNA Extraction and High-Throughput qPCR Assay

RNA extraction and high-throughput qPCR BioMark assay were performed as described previously.[65,66] The sequences of primers used, as well as their complementary probes, are available upon request or can be seen in Olagnier et al.[66]

Nuclear Protein Extraction and IRF3 DNA-Binding Activity

Nuclear and cytoplasmic proteins were isolated using the following protocol. Cells were lysed in a 400 μL solution containing 10 mM Tris, 10 mM KCl, 0.1 mM EDTA, 0.1 mM EGTA, 0.1% protease inhibitor, 1 mM DTT, 1 mM PMSF, 10 mM sodium orthovanadate, 20 mM NaF, and 40 mM β-glycerophosphate at 4°C. After 15 min, 25 μL of 10% NP40 was added, and the solution was centrifuged at maximum speed for 5 min. The supernatant was collected and kept as the cytoplasmic fraction. The pellet was washed once more in the previously mentioned lysis solution and then lysed with 25 μL of another lysis solution containing 20 mM Tris, 400 nM NaCl, 1 mM EDTA, 1 mM EGTA, 0.1% protease inhibitor, 1 mM PMSF, and 40 mM β-glycerophosphate at 4°C while vortexing. After 1 hr, the solution was centrifuged at max speed for 15 min, and the supernatant was kept as the nuclear extract. At least 10 μg of protein were loaded per gel. IRF3 TransAM ELISA kit (Active Motif) was used to evaluate IRF3 DNA-binding activity as per manufacturer's recommendations.

Plasmid Constructs

Plasmids encoding ISRE-Luc and pRLTK have been described previously.[67,68] The pP-ARE-Luciferase reporter plasmid bearing the ARE of human Nqo1 gene was a gift from Dr. Alexander Ivanov (Engelhardt Institute of Molecular Biology, Russian Academy of Sciences).[69] The Nrf2 overexpression plasmid was provided by Dr. Volker Blank (Lady Davis Institute) and was described previously.[70] The HO-1 overexpression plasmid was provided by Dr. Hyman Schipper (Lady Davis Institute) and was described previously.[71]

Luciferase Assay

For ISRE-Luc assays, experiments were performed as previously reported using the calcium phosphate transfection method.[68] For ARE luciferase assays, HEK293T cells were transfected with 50 ng of pRLTK reporter plasmid, 100 ng of pP-ARE-Luc reporter, and increasing doses of the expression plasmid encoding for Nrf2 or HO-1, along with the appropriate amount of empty vector, by the calcium phosphate transfection method. After 24 hr of transfection, luciferase activity was measured with a dual-luciferase reporter assay and a GloMax 20/20 luminometer according to the manufacturer's recommendations (Promega).

Transfection and siRNA

For siRNA experiments, A549 cells were transfected with 75 pmol of human Nrf2 (sc-37030), HO-1 (sc-35554), Atg7 (sc-41447), or control siRNA (sc-37007) diluted in Opti-MEM (Thermo Scientific) and using Lipofectamine RNAi Max as per manufacturer's instructions. A549 cells were incubated for 48 hr in the presence of the siRNA before being challenged with VSVΔ51.

Immunofluorescence

U-2 OS cells (2.5×10^5) were seeded on 1.5 mm thick, 18 by 18 mm glass coverslips (VWR); placed in each well of a 6-well plate; and treated with either vehicle [0.1% (v/v) DMSO] or SFN for 24 hr. After 20 to 24 hr, the cells were washed three times with 37°C prewarmed PBS, fixed with ice-cold methanol (−20°C) for 15 min, and permeabilized with 0.1% Triton X-100 in PBS (PBS-Tx) for a further 15 min. Next, the cells were placed in blocking solution (3% normal donkey serum in PBS-Tx) for 30 min at RT, after which they were incubated for 1–1.5 hr at RT with mouse anti-p62 (1:400, Abcam) or rabbit anti-LC3BII (1:200, Cell Signal) antibodies diluted in the blocking solution. After three washes every 5 min using PBS-Tx, the cells were incubated for 1 hr in the dark with Alexa Fluor 594 nm fluorophore-conjugated antirabbit (1:400) and Alexa Fluor 680 nm fluorophore-conjugated antimouse (1:400) secondary antibodies (Jackson ImmunoResearch) diluted in the blocking solution. After washing three times every 5 min using PBS-Tx and a further 5 min wash with PBS, the cells were incubated with DAPI (dissolved in PBS at a final concentration of 2.5 μg/mL) for 5 min at RT in the dark. The cells were quickly washed three times with PBS, mounted onto frosted glass slides (VWR) using 30 μL of ProLong gold antifade mounting medium (Invitrogen), and left to cure overnight at RT. On the following day, the cells were imaged using widefield microscope (Deltavision Elite, GE Healthcare). The data shown represent the maximum intensity projection of 25 optical sections obtained with 0.2 μm thickness per section.

In Vivo Tumor Models

TS/A Model

8-week-old-female BALB/c mice obtained from Charles River Laboratories were injected s.c. with 3×10^5 TS/A cells suspended in 100 μL PBS (n = 7 animals per group). Approximately 7 days later, when tumors became palpable (100–150 mm^3), tumors were injected with 2×10^7 PFU of VSVΔ51-GFP (in 75 μL of PBS) on day 0 and day 4. SFN at 10 mg/kg was administered i.p. 1 day before the first VSVΔ51 injection and then on days 1, 4, and 6 following the first VSVΔ51 administration. Tumors sizes were measured at the indicated time points using an electronic caliper, and tumor volume was calculated as length $\times$ $(width)^2/2$. To account for variable tumor size at the beginning of the experiment, tumor volume was normalized to its initial volume on day 0 and presented as a percentage. For survival studies, mice were euthanized when tumors had reached 2,000 mm^3 or presented evidence of necrosis or sepsis at the tumor site. Log rank (Mantel-Cox) tests were performed on Kaplan-Meier survival graphs using Prism 5 (GraphPad). To assess for the safety of the combinatorial treatment, mice were weighed every 2 days until day 8.

PC-3 Model

10-week-old-male athymic nude mice obtained from Charles River Laboratories were injected s.c. with 5×10^6 human PC-3 cells (n = 7 animals per group). Four weeks later, when tumors reached 100–150 mm^3, mice were treated intratumorally (i.t.) with

VSVΔ51-GFP (2×10^7) on days 0, 3, 6, 9, and 12 and i.p. every day with SFN (10 mg/kg) from day −1 to day 10. Tumor sizes were measured using an electronic caliper. Tumor volume and survival were determined as described previously. All experiments were performed in accordance with the Lady Davis Institute-McGill University Animal Care and Veterinary Services guidelines under protocol 2007-5212.

Statistical Analysis

Values were expressed as the mean ± SEM. Graphs and statistics were computed using Microsoft Excel or GraphPad Prism 5. An unpaired, two-tailed Student's t test was used to determine significance of the difference between the control and each experimental condition. The p values of less than 0.05 were considered statistically significant: ***$p < 0.001$, **$p < 0.01$, and *$p < 0.05$. The log rank (Mantel-Cox) test was used to determine significant differences in plots for mice survival studies. A two-way ANOVA analysis was used to calculate significant differences in plots for tumor growth studies.

SUPPLEMENTAL INFORMATION

Supplemental Information includes seven figures and can be found with this article online at http://dx.doi.org/10.1016/j.ymthe.2017.04.022.

AUTHOR CONTRIBUTIONS

D.O., J.H., and R.L. designed experiments and managed the project. D.O., R.R.L., and J.H. wrote the manuscript. D.O. performed the experiments and assembled the figures. R.R.L., S.B.H., A.S., Y.L., S.D.N., M.F., Y.J., C.C., V.B., M.L.-G., and E.V.K. performed the experiments. S.B.H., A.S., C.C., V.B., M.L.-G., E.V.K., A.T.D.-K., and R.L. edited the manuscript.

CONFLICTS OF INTEREST

The authors declare no competing financial interests and no conflict of interest

ACKNOWLEDGMENTS

The authors thank Alexander Ivanov, Josie Ursini-Siegel, Hyman Schipper, Volker Blank, and Mark Wainberg for reagents used in this study. This research work was supported by a Canadian Institutes of Health Research grant (MOP130401) and a Prostate Cancer Research Foundation of Canada grant (D2013-23) to R.L. and J.H., grants from Fondazione Cenci Bolognetti, the NIH (1R21CA192185- 01) and the Italian Association for Cancer Research (AIRC-AG16901) to J.H., and grants from Cancer Research UK (C20953/A18644) and the BBSRC (BB/L01923X/1) to A.T.D.-K. D.O. was supported by a Peter Quinlan McGill Postdoctoral Fellowship and a Canadian Cancer Society Travel Award grant. A.S. was supported by a Fond de la Recherche Sante Quebec (FRSQ) Fellowship award. The authors declare that the data supporting the findings of this study are available within the paper and can be made available from the corresponding authors upon reasonable request.

REFERENCES

1. Ilkow, C.S., Swift, S.L., Bell, J.C., and Diallo, J.S. (2014). From scourge to cure: tumour-selective viral pathogenesis as a new strategy against cancer. PLoS Pathog. *10*, e1003836.
2. Breitbach, C.J., Lichty, B.D., and Bell, J.C. (2016). Oncolytic viruses: therapeutics with an identity crisis. EBioMedicine *9*, 31–36.
3. Swift, S.L., and Stojdl, D.F. (2016). Big data offers novel insights for oncolytic virus immunotherapy. Viruses *8*, 45.
4. Lichty, B.D., Breitbach, C.J., Stojdl, D.F., and Bell, J.C. (2014). Going viral with cancer immunotherapy. Nat. Rev. Cancer *14*, 559–567.
5. Rehman, H., Silk, A.W., Kane, M.P., and Kaufman, H.L. (2016). Into the clinic: talimogene laherparepvec (T-VEC), a first-in-class intratumoral oncolytic viral therapy. J. Immunother. Cancer *4*, 53.
6. Russell, S.J., Peng, K.W., and Bell, J.C. (2012). Oncolytic virotherapy. Nat. Biotechnol. *30*, 658–670.
7. Altomonte, J., Wu, L., Chen, L., Meseck, M., Ebert, O., García-Sastre, A., Fallon, J., and Woo, S.L. (2008). Exponential enhancement of oncolytic vesicular stomatitis virus potency by vector-mediated suppression of inflammatory responses in vivo. Mol. Ther. *16*, 146–153.
8. Arulanandam, R., Batenchuk, C., Varette, O., Zakaria, C., Garcia, V., Forbes, N.E., Davis, C., Krishnan, R., Karmacharya, R., Cox, J., et al. (2015). Microtubule disruption synergizes with oncolytic virotherapy by inhibiting interferon translation and potentiating bystander killing. Nat. Commun. *6*, 6410.
9. Ben Yebdri, F., Van Grevenynghe, J., Tang, V.A., Goulet, M.L., Wu, J.H., Stojdl, D.F., Hiscott, J., and Lin, R. (2013). Triptolide-mediated inhibition of interferon signaling enhances vesicular stomatitis virus-based oncolysis. Mol. Ther. *21*, 2043–2053.
10. Nguyên, T.L., Abdelbary, H., Arguello, M., Breitbach, C., Leveille, S., Diallo, J.S., Yasmeen, A., Bismar, T.A., Kirn, D., Falls, T., et al. (2008). Chemical targeting of the innate antiviral response by histone deacetylase inhibitors renders refractory cancers sensitive to viral oncolysis. Proc. Natl. Acad. Sci. USA *105*, 14981–14986.
11. Ottolino-Perry, K., Diallo, J.S., Lichty, B.D., Bell, J.C., and McCart, J.A. (2010). Intelligent design: combination therapy with oncolytic viruses. Mol. Ther. *18*, 251–263.
12. Shulak, L., Beljanski, V., Chiang, C., Dutta, S.M., Van Grevenynghe, J., Belgnaoui, S.M., Nguyên, T.L., Di Lenardo, T., Semmes, O.J., Lin, R., and Hiscott, J. (2014). Histone deacetylase inhibitors potentiate vesicular stomatitis virus oncolysis in prostate cancer cells by modulating NF-κB-dependent autophagy. J. Virol. *88*, 2927–2940.
13. Dobson, C.C., Naing, T., Beug, S.T., Faye, M.D., Chabot, J., St-Jean, M., Walker, D.E., LaCasse, E.C., Stojdl, D.F., Korneluk, R.G., and Holcik, M. (2017). Oncolytic virus synergizes with Smac mimetic compounds to induce rhabdomyosarcoma cell death in a syngeneic murine model. Oncotarget *8*, 3495–3508.
14. Marchini, A., Scott, E.M., and Rommelaere, J. (2016). Overcoming barriers in oncolytic virotherapy with HDAC inhibitors and immune checkpoint blockade. Viruses *8*, 9.
15. Breitbach, C.J., Reid, T., Burke, J., Bell, J.C., and Kirn, D.H. (2010). Navigating the clinical development landscape for oncolytic viruses and other cancer therapeutics: no shortcuts on the road to approval. Cytokine Growth Factor Rev. *21*, 85–89.
16. Rowan, K. (2010). Oncolytic viruses move forward in clinical trials. J. Natl. Cancer Inst. *102*, 590–595.
17. Stojdl, D.F., Lichty, B., Knowles, S., Marius, R., Atkins, H., Sonenberg, N., and Bell, J.C. (2000). Exploiting tumor-specific defects in the interferon pathway with a previously unknown oncolytic virus. Nat. Med. *6*, 821–825.
18. Stojdl, D.F., Lichty, B.D., tenOever, B.R., Paterson, J.M., Power, A.T., Knowles, S., Marius, R., Reynard, J., Poliquin, L., Atkins, H., et al. (2003). VSV strains with defects in their ability to shutdown innate immunity are potent systemic anti-cancer agents. Cancer Cell *4*, 263–275.
19. Samuel, S., Tumilasci, V.F., Oliere, S., Nguyên, T.L., Shamy, A., Bell, J., and Hiscott, J. (2010). VSV oncolysis in combination with the BCL-2 inhibitor obatoclax overcomes apoptosis resistance in chronic lymphocytic leukemia. Mol. Ther. *18*, 2094–2103.
20. Nguyen, T., Nioi, P., and Pickett, C.B. (2009). The Nrf2-antioxidant response element signaling pathway and its activation by oxidative stress. J. Biol. Chem. *284*, 13291–13295.

21. Baird, L., Llères, D., Swift, S., and Dinkova-Kostova, A.T. (2013). Regulatory flexibility in the Nrf2-mediated stress response is conferred by conformational cycling of the Keap1-Nrf2 protein complex. Proc. Natl. Acad. Sci. USA *110*, 15259–15264.

22. Jaramillo, M.C., and Zhang, D.D. (2013). The emerging role of the Nrf2-Keap1 signaling pathway in cancer. Genes Dev. *27*, 2179–2191.

23. Greaney, A.J., Maier, N.K., Leppla, S.H., and Moayeri, M. (2016). Sulforaphane inhibits multiple inflammasomes through an Nrf2-independent mechanism. J. Leukoc. Biol. *99*, 189–199.

24. Qi, T., Xu, F., Yan, X., Li, S., and Li, H. (2016). Sulforaphane exerts anti-inflammatory effects against lipopolysaccharide-induced acute lung injury in mice through the Nrf2/ARE pathway. Int. J. Mol. Med. *37*, 182–188.

25. Traka, M., Gasper, A.V., Melchini, A., Bacon, J.R., Needs, P.W., Frost, V., Chantry, A., Jones, A.M., Ortori, C.A., Barrett, D.A., et al. (2008). Broccoli consumption interacts with GSTM1 to perturb oncogenic signalling pathways in the prostate. PLoS ONE *3*, e2568.

26. Alumkal, J.J., Slottke, R., Schwartzman, J., Cherala, G., Munar, M., Graff, J.N., Beer, T.M., Ryan, C.W., Koop, D.R., Gibbs, A., et al. (2015). A phase II study of sulforaphane-rich broccoli sprout extracts in men with recurrent prostate cancer. Invest. New Drugs *33*, 480–489.

27. Cipolla, B.G., Mandron, E., Lefort, J.M., Coadou, Y., Della Negra, E., Corbel, L., Le Scodan, R., Azzouzi, A.R., and Mottet, N. (2015). Effect of sulforaphane in men with biochemical recurrence after radical prostatectomy. Cancer Prev. Res. (Phila.) *8*, 712–719.

28. van Die, M.D., Bone, K.M., Emery, J., Williams, S.G., Pirotta, M.V., and Paller, C.J. (2016). Phytotherapeutic interventions in the management of biochemically recurrent prostate cancer: a systematic review of randomised trials. BJU Int. *117* (*Suppl 4*), 17–34.

29. Furfaro, A.L., Traverso, N., Domenicotti, C., Piras, S., Moretta, L., Marinari, U.M., Pronzato, M.A., and Nitti, M. (2016). The Nrf2/HO-1 axis in cancer cell growth and chemoresistance. Oxid. Med. Cell. Longev. *2016*, 1958174.

30. Jeong, Y., Hoang, N.T., Lovejoy, A., Stehr, H., Newman, A.M., Gentles, A.J., Kong, W., Truong, D., Martin, S., Chaudhuri, A., et al. (2017). Role of KEAP1/NRF2 and TP53 mutations in lung squamous cell carcinoma development and radiation resistance. Cancer Discov. *7*, 86–101.

31. Singh, A., Misra, V., Thimmulappa, R.K., Lee, H., Ames, S., Hoque, M.O., Herman, J.G., Baylin, S.B., Sidransky, D., Gabrielson, E., et al. (2006). Dysfunctional KEAP1-NRF2 interaction in non-small-cell lung cancer. PLoS Med. *3*, e420.

32. Lee, Y.J., Jeong, H.Y., Kim, Y.B., Lee, Y.J., Won, S.Y., Shim, J.H., Cho, M.K., Nam, H.S., and Lee, S.H. (2012). Reactive oxygen species and PI3K/Akt signaling play key roles in the induction of Nrf2-driven heme oxygenase-1 expression in sulforaphane-treated human mesothelioma MSTO-211H cells. Food Chem. Toxicol. *50*, 116–123.

33. Lee, Y.J., and Lee, S.H. (2011). Sulforaphane induces antioxidative and antiproliferative responses by generating reactive oxygen species in human bronchial epithelial BEAS-2B cells. J. Korean Med. Sci. *26*, 1474–1482.

34. Dinkova-Kostova, A.T., Holtzclaw, W.D., Cole, R.N., Itoh, K., Wakabayashi, N., Katoh, Y., Yamamoto, M., and Talalay, P. (2002). Direct evidence that sulfhydryl groups of Keap1 are the sensors regulating induction of phase 2 enzymes that protect against carcinogens and oxidants. Proc. Natl. Acad. Sci. USA *99*, 11908–11913.

35. Biswas, C., Shah, N., Muthu, M., La, P., Fernando, A.P., Sengupta, S., Yang, G., and Dennery, P.A. (2014). Nuclear heme oxygenase-1 (HO-1) modulates subcellular distribution and activation of Nrf2, impacting metabolic and anti-oxidant defenses. J. Biol. Chem. *289*, 26882–26894.

36. Herman-Antosiewicz, A., Johnson, D.E., and Singh, S.V. (2006). Sulforaphane causes autophagy to inhibit release of cytochrome C and apoptosis in human prostate cancer cells. Cancer Res. *66*, 5828–5835.

37. Jounai, N., Takeshita, F., Kobiyama, K., Sawano, A., Miyawaki, A., Xin, K.Q., Ishii, K.J., Kawai, T., Akira, S., Suzuki, K., and Okuda, K. (2007). The Atg5 Atg12 conjugate associates with innate antiviral immune responses. Proc. Natl. Acad. Sci. USA *104*, 14050–14055.

38. Pajares, M., Jiménez-Moreno, N., García-Yagüe, A.J., Escoll, M., de Ceballos, M.L., Van Leuven, F., Rábano, A., Yamamoto, M., Rojo, A.I., and Cuadrado, A. (2016). Transcription factor NFE2L2/NRF2 is a regulator of macroautophagy genes. Autophagy *12*, 1902–1916.

39. Hiscott, J. (2007). Triggering the innate antiviral response through IRF-3 activation. J. Biol. Chem. *282*, 15325–15329.

40. Zhang, Y., Talalay, P., Cho, C.G., and Posner, G.H. (1992). A major inducer of anticarcinogenic protective enzymes from broccoli: isolation and elucidation of structure. Proc. Natl. Acad. Sci. USA *89*, 2399–2403.

41. Fahey, J.W., Zhang, Y., and Talalay, P. (1997). Broccoli sprouts: an exceptionally rich source of inducers of enzymes that protect against chemical carcinogens. Proc. Natl. Acad. Sci. USA *94*, 10367–10372.

42. Lenzi, M., Fimognari, C., and Hrelia, P. (2014). Sulforaphane as a promising molecule for fighting cancer. Cancer Treat. Res. *159*, 207–223.

43. Dinkova-Kostova, A.T., Fahey, J.W., Kostov, R.V., and Kensler, T.W. (2017). KEAP1 and done? Targeting the NRF2 pathway with sulforaphane. Trends Food Sci. Technol., Published online February 16, 2017. http://dx.doi.org/10.1016/j.tifs.2017.02.002.

44. Singh, S.V., Srivastava, S.K., Choi, S., Lew, K.L., Antosiewicz, J., Xiao, D., Zeng, Y., Watkins, S.C., Johnson, C.S., Trump, D.L., et al. (2005). Sulforaphane-induced cell death in human prostate cancer cells is initiated by reactive oxygen species. J. Biol. Chem. *280*, 19911–19924.

45. Hu, C., Eggler, A.L., Mesecar, A.D., and van Breemen, R.B. (2011). Modification of keap1 cysteine residues by sulforaphane. Chem. Res. Toxicol. *24*, 515–521.

46. Zhang, D.D., and Hannink, M. (2003). Distinct cysteine residues in Keap1 are required for Keap1-dependent ubiquitination of Nrf2 and for stabilization of Nrf2 by chemopreventive agents and oxidative stress. Mol. Cell. Biol. *23*, 8137–8151.

47. McMahon, M., Lamont, D.J., Beattie, K.A., and Hayes, J.D. (2010). Keap1 perceives stress via three sensors for the endogenous signaling molecules nitric oxide, zinc, and alkenals. Proc. Natl. Acad. Sci. USA *107*, 18838–18843.

48. Alexander, A., Cai, S.L., Kim, J., Nanez, A., Sahin, M., MacLean, K.H., Inoki, K., Guan, K.L., Shen, J., Person, M.D., et al. (2010). ATM signals to TSC2 in the cytoplasm to regulate mTORC1 in response to ROS. Proc. Natl. Acad. Sci. USA *107*, 4153–4158.

49. Otterbein, L.E., Hedblom, A., Harris, C., Csizmadia, E., Gallo, D., and Wegiel, B. (2011). Heme oxygenase-1 and carbon monoxide modulate DNA repair through ataxia-telangiectasia mutated (ATM) protein. Proc. Natl. Acad. Sci. USA *108*, 14491–14496.

50. Alain, T., Lun, X., Martineau, Y., Sean, P., Pulendran, B., Petroulakis, E., Zemp, F.J., Lemay, C.G., Roy, D., Bell, J.C., et al. (2010). Vesicular stomatitis virus oncolysis is potentiated by impairing mTORC1-dependent type I IFN production. Proc. Natl. Acad. Sci. USA *107*, 1576–1581.

51. Yang, G., Lee, H.E., and Lee, J.Y. (2016). A pharmacological inhibitor of NLRP3 inflammasome prevents non-alcoholic fatty liver disease in a mouse model induced by high fat diet. Sci. Rep. *6*, 24399.

52. Liu, Z., Ravindranathan, R., Kalinski, P., Guo, Z.S., and Bartlett, D.L. (2017). Rational combination of oncolytic vaccinia virus and PD-L1 blockade works synergistically to enhance therapeutic efficacy. Nat. Commun. *8*, 14754.

53. Simpson, G.R., Relph, K., Harrington, K., Melcher, A., and Pandha, H. (2016). Cancer immunotherapy via combining oncolytic virotherapy with chemotherapy: recent advances. Oncolytic Virother. *5*, 1–13.

54. Rajani, K.R., and Vile, R.G. (2015). Harnessing the power of onco-immunotherapy with checkpoint inhibitors. Viruses *7*, 5889–5901.

55. Bridle, B.W., Clouthier, D., Zhang, L., Pol, J., Chen, L., Lichty, B.D., Bramson, J.L., and Wan, Y. (2013). Oncolytic vesicular stomatitis virus quantitatively and qualitatively improves primary CD8(+) T-cell responses to anticancer vaccines. OncoImmunology *2*, e26013.

56. Bridle, B.W., Chen, L., Lemay, C.G., Diallo, J.S., Pol, J., Nguyen, A., Capretta, A., He, R., Bramson, J.L., Bell, J.C., et al. (2013). HDAC inhibition suppresses primary immune responses, enhances secondary immune responses, and abrogates autoimmunity during tumor immunotherapy. Mol. Ther. *21*, 887–894.

57. Lemay, C.G., Keller, B.A., Edge, R.E., Abei, M., and Bell, J.C. (2017). Oncolytic viruses: the best is yet to come. Curr. Cancer Drug Targets, Published online February 6, 2017. http://dx.doi.org/10.2174/1568009617666170206111609.

58. Kallifatidis, G., Labsch, S., Rausch, V., Mattern, J., Gladkich, J., Moldenhauer, G., Büchler, M.W., Salnikov, A.V., and Herr, I. (2011). Sulforaphane increases drug-mediated cytotoxicity toward cancer stem-like cells of pancreas and prostate. Mol. Ther. *19*, 188–195.

59. Erzinger, M.M., Bovet, C., Hecht, K.M., Senger, S., Winiker, P., Sobotzki, N., Cristea, S., Beerenwinkel, N., Shay, J.W., Marra, G., et al. (2016). Sulforaphane preconditioning sensitizes human colon cancer cells towards the bioreductive anticancer prodrug PR-104A. PLoS ONE *11*, e0150219.

60. Higgins, L.G., and Hayes, J.D. (2011). The cap'n'collar transcription factor Nrf2 mediates both intrinsic resistance to environmental stressors and an adaptive response elicited by chemopreventive agents that determines susceptibility to electrophilic xenobiotics. Chem. Biol. Interact. *192*, 37–45.

61. Wakabayashi, N., Itoh, K., Wakabayashi, J., Motohashi, H., Noda, S., Takahashi, S., Imakado, S., Kotsuji, T., Otsuka, F., Roop, D.R., et al. (2003). Keap1-null mutation leads to postnatal lethality due to constitutive Nrf2 activation. Nat. Genet. *35*, 238–245.

62. Kovac, S., Angelova, P.R., Holmström, K.M., Zhang, Y., Dinkova-Kostova, A.T., and Abramov, A.Y. (2015). Nrf2 regulates ROS production by mitochondria and NADPH oxidase. Biochim. Biophys. Acta *1850*, 794–801.

63. Kuma, A., Hatano, M., Matsui, M., Yamamoto, A., Nakaya, H., Yoshimori, T., Ohsumi, Y., Tokuhisa, T., and Mizushima, N. (2004). The role of autophagy during the early neonatal starvation period. Nature *432*, 1032–1036.

64. Olagnier, D., Scholte, F.E., Chiang, C., Albulescu, I.C., Nichols, C., He, Z., Lin, R., Snijder, E.J., van Hemert, M.J., and Hiscott, J. (2014). Inhibition of dengue and chikungunya virus infections by RIG-I-mediated type I interferon-independent stimulation of the innate antiviral response. J. Virol. *88*, 4180–4194.

65. Chiang, C., Beljanski, V., Yin, K., Olagnier, D., Ben Yebdri, F., Steel, C., Goulet, M.L., DeFilippis, V.R., Streblow, D.N., Haddad, E.K., et al. (2015). Sequence-specific modifications enhance the broad-spectrum antiviral response activated by RIG-I agonists. J. Virol. *89*, 8011–8025.

66. Olagnier, D., Peri, S., Steel, C., van Montfoort, N., Chiang, C., Beljanski, V., Slifker, M., He, Z., Nichols, C.N., Lin, R., et al. (2014). Cellular oxidative stress response controls the antiviral and apoptotic programs in dengue virus-infected dendritic cells. PLoS Pathog. *10*, e1004566.

67. Belgnaoui, S.M., Paz, S., Samuel, S., Goulet, M.L., Sun, Q., Kikkert, M., Iwai, K., Dikic, I., Hiscott, J., and Lin, R. (2012). Linear ubiquitination of NEMO negatively regulates the interferon antiviral response through disruption of the MAVS-TRAF3 complex. Cell Host Microbe *12*, 211–222.

68. Zhao, T., Yang, L., Sun, Q., Arguello, M., Ballard, D.W., Hiscott, J., and Lin, R. (2007). The NEMO adaptor bridges the nuclear factor-kappaB and interferon regulatory factor signaling pathways. Nat. Immunol. *8*, 592–600.

69. Ivanov, A.V., Smirnova, O.A., Ivanova, O.N., Masalova, O.V., Kochetkov, S.N., and Isaguliants, M.G. (2011). Hepatitis C virus proteins activate NRF2/ARE pathway by distinct ROS-dependent and independent mechanisms in HUH7 cells. PLoS ONE *6*, e24957.

70. Massrieh, W., Derjuga, A., and Blank, V. (2006). Induction of endogenous Nrf2/small maf heterodimers by arsenic-mediated stress in placental choriocarcinoma cells. Antioxid. Redox Signal. *8*, 53–59.

71. Song, W., Su, H., Song, S., Paudel, H.K., and Schipper, H.M. (2006). Over-expression of heme oxygenase-1 promotes oxidative mitochondrial damage in rat astroglia. J. Cell. Physiol. *206*, 655–663.

YMTHE, Volume *25*

Supplemental Information

Activation of Nrf2 Signaling Augments Vesicular Stomatitis Virus Oncolysis via Autophagy-Driven Suppression of Antiviral Immunity

David Olagnier, Rassin R. Lababidi, Samar Bel Hadj, Alexandre Sze, Yiliu Liu, Sharadha Dayalan Naidu, Matteo Ferrari, Yuan Jiang, Cindy Chiang, Vladimir Beljanski, Marie-Line Goulet, Elena V. Knatko, Albena T. Dinkova-Kostova, John Hiscott, and Rongtuan Lin

Supplementary Materials

Supplemental Figure Legends

Figure S1. Sulforaphane potentiates VSVΔ51 infectivity in various cancer cell lines. Human prostate DU145, human breast MDA-MB, and murine breast TS-A were pretreated for 24h with SFN and then challenged with VSVΔ51. 24 hours later, viral infectivity was determined by flow cytometry. Data are the means ± SEM from at least two experiments performed in duplicate or triplicate on each cell line.

Figure S2. Sulforaphane treatment does not affect PC-3 viability *in vitro.* Cytotoxic effect of SFN alone was tested in PC-3 cells by treating the cells with increasing concentrations of SFN for 24h and 48h. **(a)** SFN toxicity on PC-3 cells was visualized 48h after treatment by light microscopy. **(b-c)** The percentage of viable and apoptotic cells was assessed by flow cytometry using a 7AAD/annexin-V staining as previously described.

Figure S3. Sulforaphane does not increase VSVΔ51 infectivity and oncolysis in non-cancerous human fibroblasts. (a-b) Human prostate PC-3 cancer cells and human MRC-5 normal fibroblasts were pre-treated with SFN (15µM) for 24h and were subsequently infected with VSVΔ51-GFP (MOI 0.1). Infectivity **(a)** and oncolytic activity **(b)** were determined by flow cytometry at 48h post-infection. Data are the means ± SEM from two independent experiments performed in quadruplicate for MRC-5 and triplicate for PC-3.

Figure S4. **Diethylmaleate stimulates Nrf2 promoter activity and increases VSVΔ51 infectivity in resistant prostate cancer cells. (a)** HEK 293T cells were pre-treated for 24h with increasing doses of diethylmaleate (DEM) and ARE promoter activity was assessed using a luciferase assay. **(b)** PC-3 cells were pre-treated with sulforaphane (SFN) (20µM) or diethylmaleate (100µM) for 24h and were subsequently infected with VSVΔ51-GFP (MOI 0.1 or 1). Viral infectivity was determined by flow cytometry based on GFP expression.

Figure S5. SFN activates Nrf2 and increases VSVΔ51 infectivity independently of ROS. (a) ROS generation in SFN-treated PC-3 cells was monitored by flow cytometry using CM-H2DCFDA (1µM) at 4 and 24h following treatment. H_2O_2 (500µM) and pyocyanin (100µM) were used as positive internal controls of ROS accumulation. **(b)** ROS generation was also monitored 24h after treatment with H_2O_2 (500µM) or pyocyanin (100µM) in presence or absence of L-NAC (2mM) and SFN (20µM). **(c)** HEK 293T cells were pre-treated with L-NAC (10mM) for 30 min which was subsequently washed away. Cells were then treated with SFN (10µM) for 18h. ARE promoter activity was assessed using a luciferase assay. **(d)** PC-3 cells were pre-treated with L-NAC (10mM) for 30 min. L-NAC was removed and cells stimulated or not with SFN (20µM) for 24h prior to challenge with VSVΔ51 (MOI 1) for 24h. Viral infectivity was determined by flow cytometry based on GFP expression.

Figure S6. IRF3 does not bind to DNA following SFN stimulation in VSVΔ51-infected PC-3 cells. IRF3-nuclear binding activity was assessed using an IRF3-binding ELISA TransAM kit after treatment of PC-3 cells with SFN (20µM) for 24h and infection with VSVΔ51 (MOI 1) for 8 hrs. The data are expressed as relative arbitrary units. Data are the means from two independent experiments.

Figure S7. Abrogating autophagy reduces VSVΔ51 infection and replication in A549 lung cancer cells. A549 cells were transfected with control or Atg7 siRNA and 48h later were challenged with VSVΔ51-GFP (MOI 0.01) for an extra day. (**a**) VSVΔ51 infection was determined by flow cytometry and pictures were taken using the ZOE fluorescent cell imager. Data are the means± SEM from three independent experiments. (**b**) Viral replication was assessed by plaque assay. Data are the means± SEM from one experiment performed in triplicate.

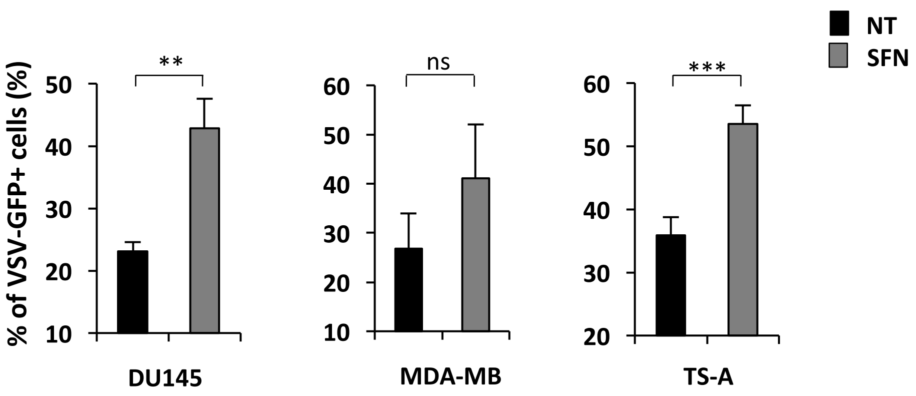

NT
SFN
% of VSV-GFP+ cells (%)
**
ns
***
50
40
30
20
10
60
50
40
30
20
10
60
50
40
30
20
DU145
MDA-MB
TS-A

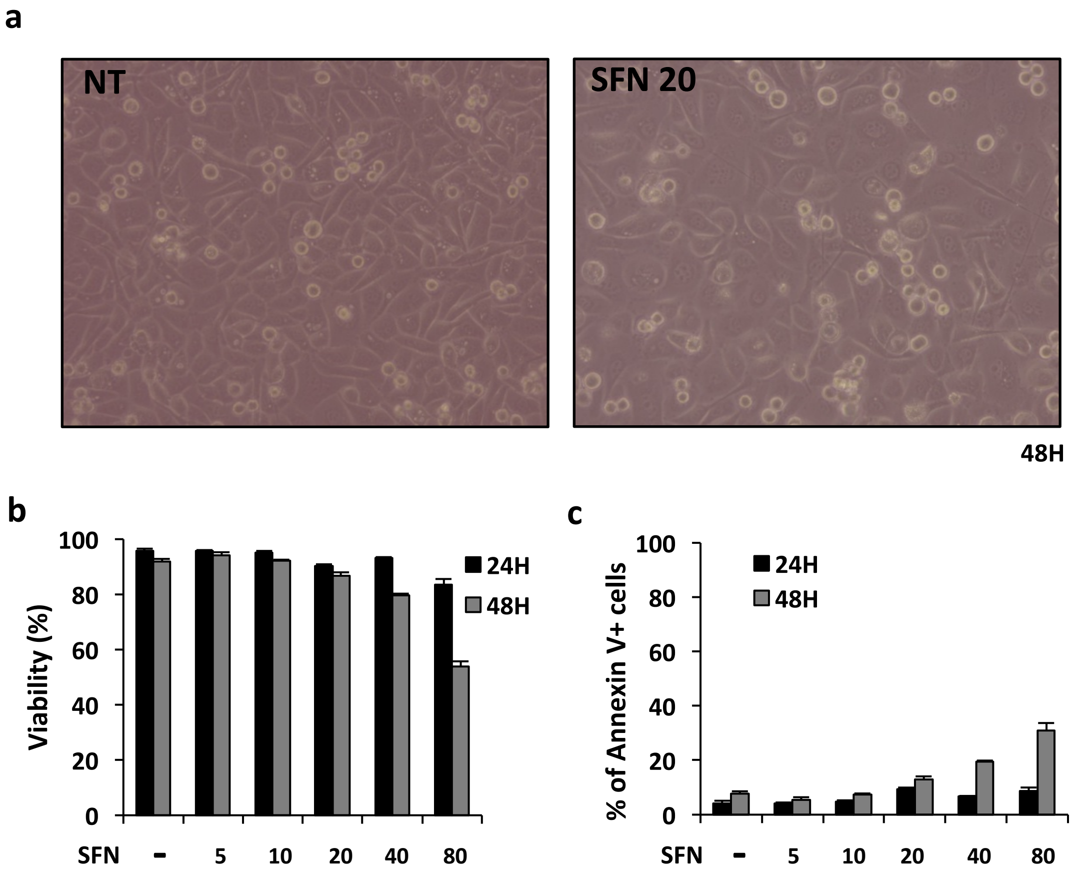

a
NT
SFN 20
48H
b
Viability (%)
24H
48H
SFN
-
5
10
20
40
80
c
% of Annexin V+ cells
24H
48H
SFN
-
5
10
20
40
80

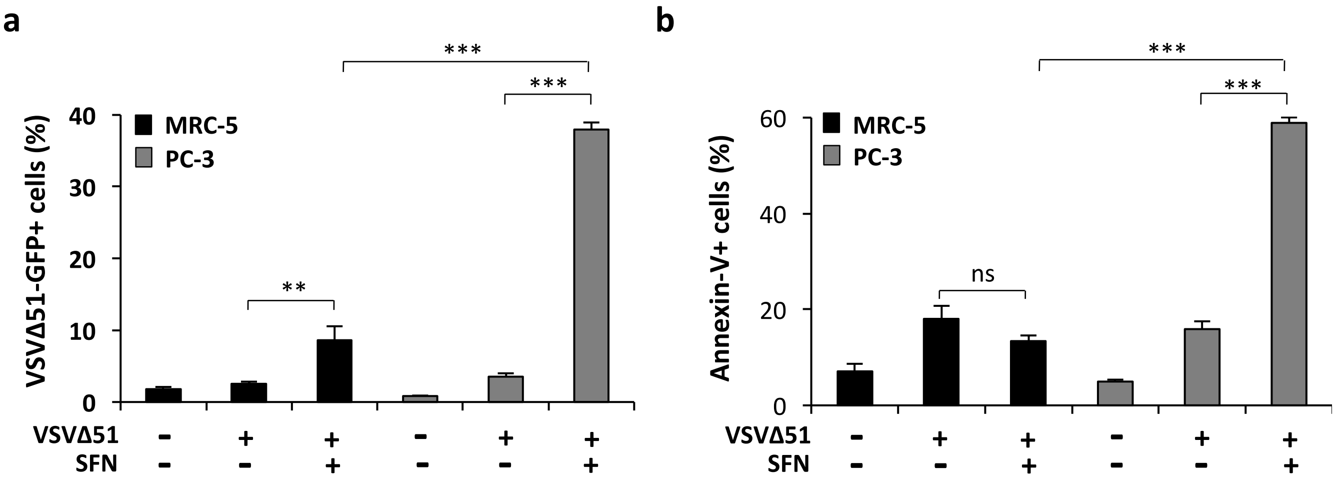
a
VSVΔ51-GFP+ cells (%)
MRC-5
PC-3
0
10
20
30
40
**
***
***
VSVΔ51
SFN
b
Annexin-V+ cells (%)
MRC-5
PC-3
0
20
40
60
ns
***
***
VSVΔ51
SFN

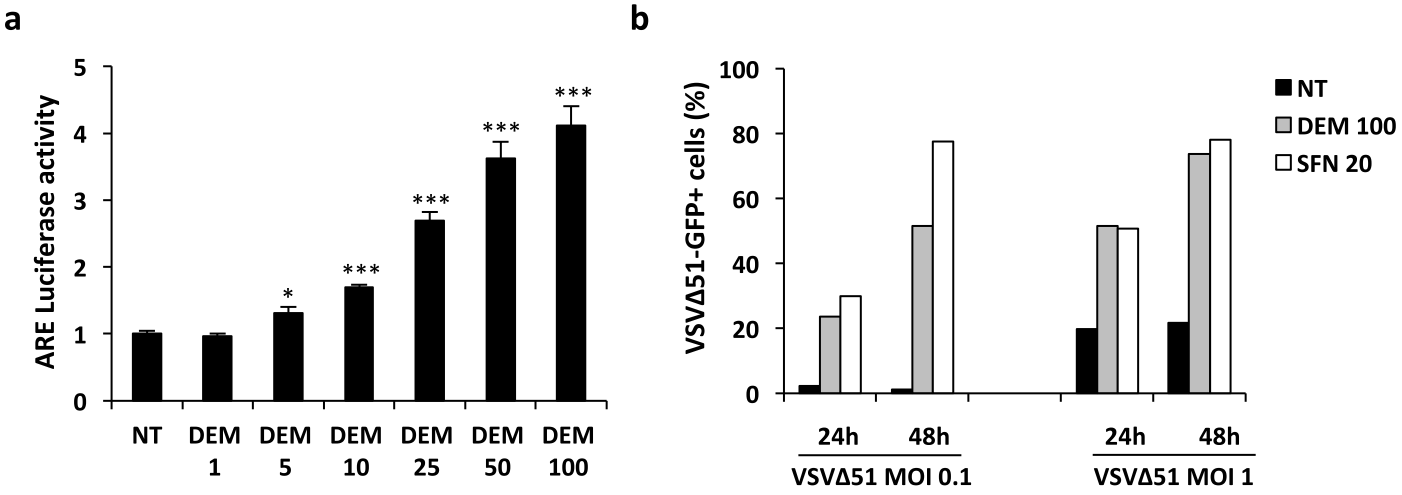
a
ARE Luciferase activity
5
4
3
2
1
0
NT
DEM 1
DEM 5
DEM 10
DEM 25
DEM 50
DEM 100
*
***
***
***
***
b
VSVΔ51-GFP+ cells (%)
100
80
60
40
20
0
NT
DEM 100
SFN 20
24h
48h
VSVΔ51 MOI 0.1
24h
48h
VSVΔ51 MOI 1

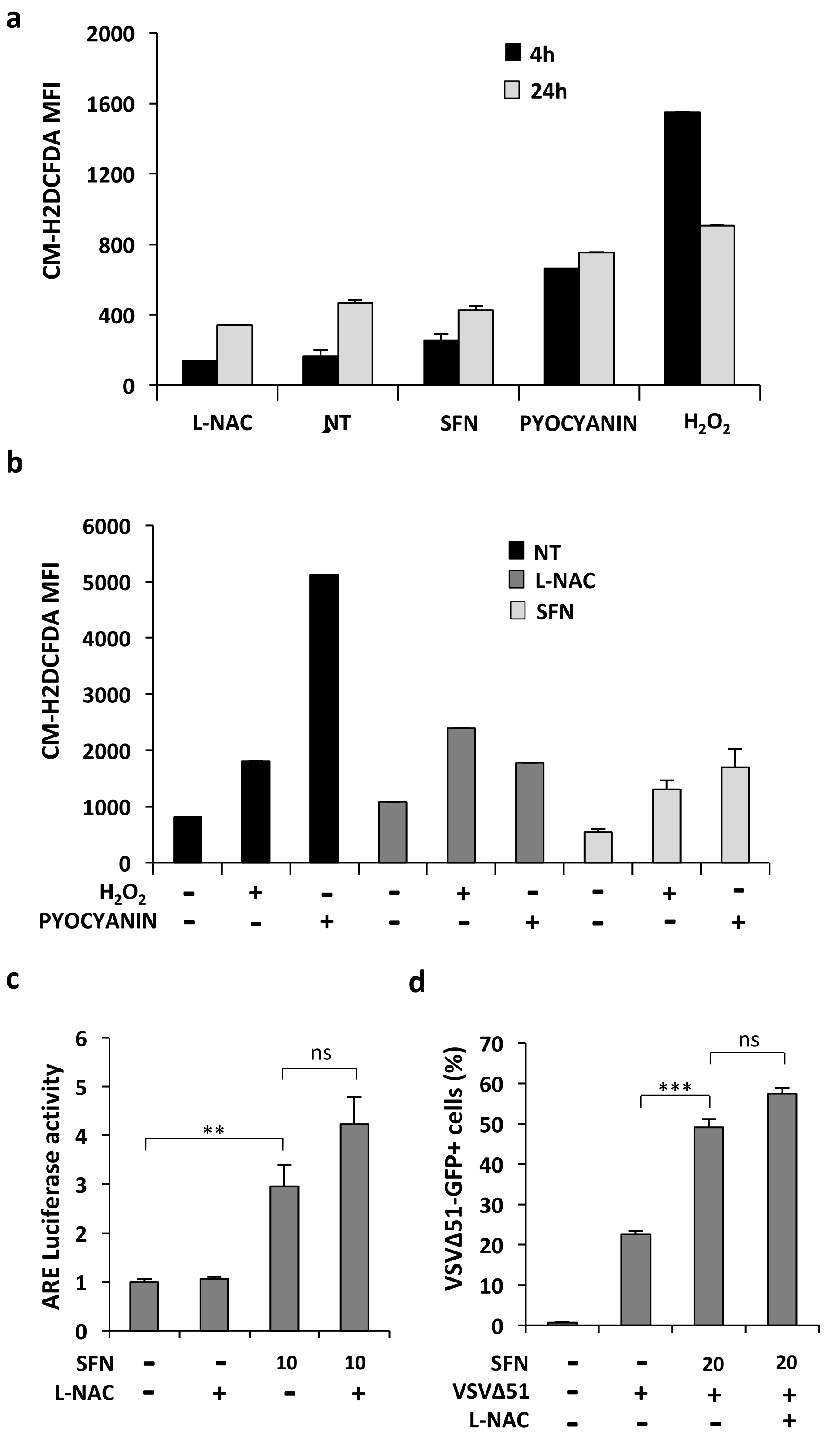
a
CM-H2DCFDA MFI
2000
1600
1200
800
400
0
4h
24h
L-NAC
NT
SFN
PYOCYANIN
H_2O_2
b
CM-H2DCFDA MFI
6000
5000
4000
3000
2000
1000
0
NT
L-NAC
SFN
H_2O_2 - + - - + - - + -
PYOCYANIN - - + - - + - - +
c
ARE Luciferase activity
6
5
4
3
2
1
0
ns
**
SFN - - 10 10
L-NAC - + - +
d
VSVΔ51-GFP+ cells (%)
70
60
50
40
30
20
10
0
ns
***
SFN - - 20 20
VSVΔ51 - + + +
L-NAC - - - +

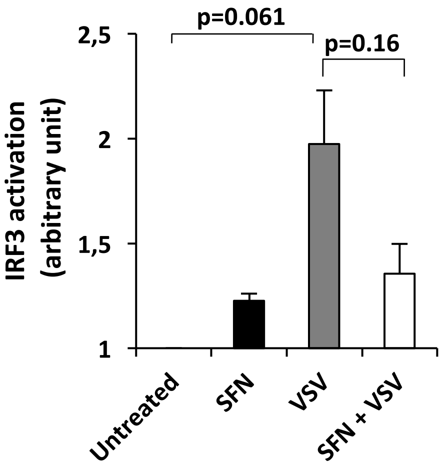
p=0.061
p=0.16
IRF3 activation
(arbitrary unit)
2,5
2
1,5
1
Untreated
SFN
VSV
SFN + VSV

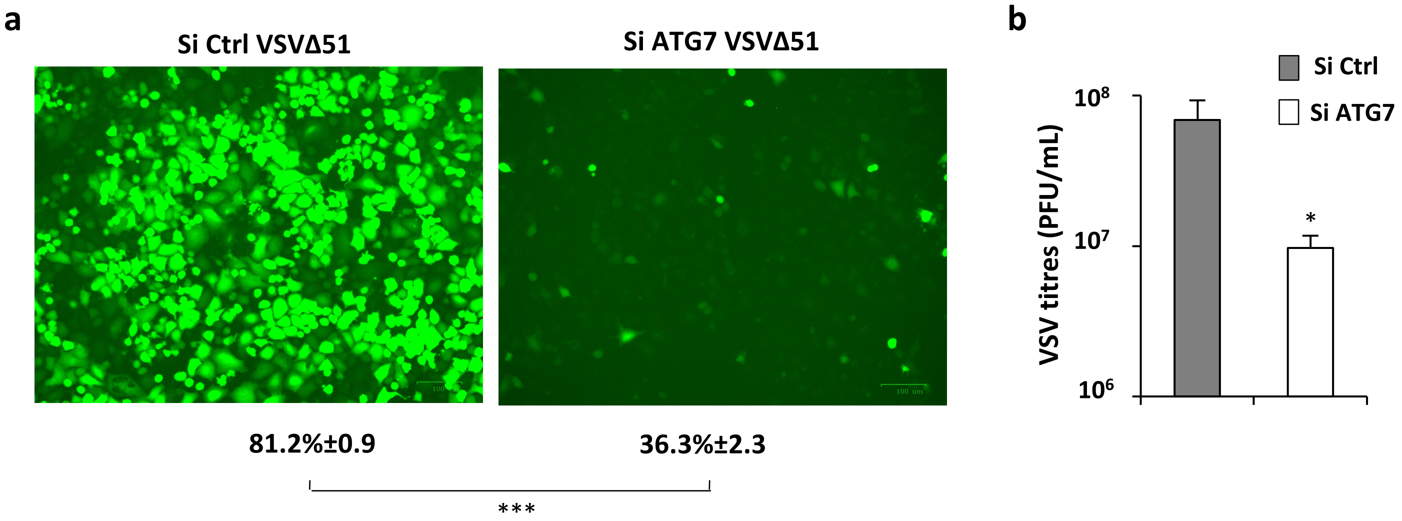
a
Si Ctrl VSVΔ51
Si ATG7 VSVΔ51
81.2%±0.9
36.3%±2.3
***
b
Si Ctrl
Si ATG7
VSV titres (PFU/mL)
10^8
10^7
10^6
*
